# RNA-activated protein cleavage with a CRISPR-associated endopeptidase

**DOI:** 10.1126/science.add7450

**Published:** 2022-11-03

**Authors:** Jonathan Strecker, F. Esra Demircioglu, David Li, Guilhem Faure, Max E. Wilkinson, Jonathan S. Gootenberg, Omar O. Abudayyeh, Hiroshi Nishimasu, Rhiannon K. Macrae, Feng Zhang

**Affiliations:** 1 Howard Hughes Medical Institute, Cambridge, MA 02139, USA; 2 Broad Institute of MIT and Harvard, Cambridge, MA 02142, USA; 3 McGovern Institute for Brain Research, Cambridge, MA 02139, USA; 4 Department of Brain and Cognitive Sciences, Cambridge, MA 02139, USA; 5 Department of Biological Engineering, Cambridge, MA 02139, USA; 6 Department of Electrical Engineering and Computer Science, Massachusetts Institute of Technology, Cambridge, MA 02139, USA; 7 Structural Biology Division, Research Center for Advanced Science and Technology, The University of Tokyo, Tokyo 153-8904, Japan; 8 Department of Chemistry and Biotechnology, Graduate School of Engineering, The University of Tokyo, Tokyo 113-8656, Japan; 9 Department of Biological Sciences, Graduate School of Science, The University of Tokyo, Tokyo 113-0033, Japan; 10 Inamori Research Institute for Science, 620 Suiginya-cho, Kyoto 600-8411, Japan

## Abstract

CRISPR-Cas systems provide adaptive immune responses in prokaryotes against foreign genetic elements through RNA-guided nuclease activity. Recently, additional genes with non-nuclease functions have been found in genetic association with CRISPR systems, suggesting there may be other RNA-guided non-nucleolytic enzymes. One such gene encodes the TPR-CHAT protease Csx29, which is associated with the CRISPR effector Cas7–11. Here, we demonstrate that this CRISPR-associated protease (CASP) exhibits programmable RNA-activated endopeptidase activity against a sigma factor inhibitor to regulate a transcriptional response. Cryo–electron microscopy of an active and substrate-bound CASP complex reveals an allosteric activation mechanism that reorganizes Csx29 catalytic residues upon target RNA binding. This work reveals an RNA-guided function in nature which can be leveraged for RNA sensing applications in vitro and in human cells.

Prokaryotes possess a multitude of defense systems against foreign genetic elements, including clustered regularly interspaced short palindromic repeats (CRISPR) and CRISPR-associated proteins (Cas) systems (1–3). While the predominant function of CRISPR-Cas systems is to provide adaptive immunity via RNA-guided DNA or RNA nuclease activity, additional proteins have been identified in genetic association with CRISPR loci (3–5). One example is that of the CRISPR-associated transposase (CAST) systems (6, 7), which perform RNA-guided DNA insertion whereby nuclease inactive CRISPR effectors guide Tn7-like mobile genetic elements to specific DNA sequences (8, 9). CAST systems have evolved on at least three separate occasions (10), highlighting the ability of diverse CRISPR effectors to acquire, or be acquired by, other bacterial enzymes. Beyond CAST systems, additional functions genetically linked to CRISPR-Cas systems are beginning to emerge, and more likely remain to be discovered and characterized.

Previous work has uncovered several RNA-targeting type III CRISPR-associated protease (CASP) systems (3, 4), including a Lon protease that responds to cyclic oligoadenylate second messengers (cA_4_) to cleave the CRISPR-T protein (11). A recently characterized subtype III-E effector Cas7–11 (12, 13) (also referred to as gRAMP) is likewise associated with a protease, a CHAT family member containing tetratricopeptide repeats (TPR-CHAT, or Csx29). In contrast to prototypical type III CRISPR systems consisting of multi-subunit Csm/Cmr complexes (14), Cas7–11 effectors contain naturally fused Cas7 and Cas11 domains (3). Members of the CHAT family of proteases harbor catalytic cysteine residues and include eukaryotic caspases involved in programmed cell death (15), and Cas7–11-Csx29 was previously hypothesized to act as a bacterial caspase and support viral immunity (12, 13). Notably, Cas7–11 and Csx29 from *Candidatus Scalindua brodae* were shown to form a stable protein complex (13), but the substrate and function of the associated protease is unknown.

Here, we determine the protein substrate, structure, and mechanism of a type III-E CRISPR-associated protease (CASP) from the marine anaerobe *Desulfonema ishimotonii*, reveal insight into its natural function in coordinating a transcriptional response to foreign genetic material, and engineer it for novel RNA sensing applications in vitro and in human cells.

## A Cas7–11-Csx29 complex cleaves the Csx30 protein

**Fig. 1.**
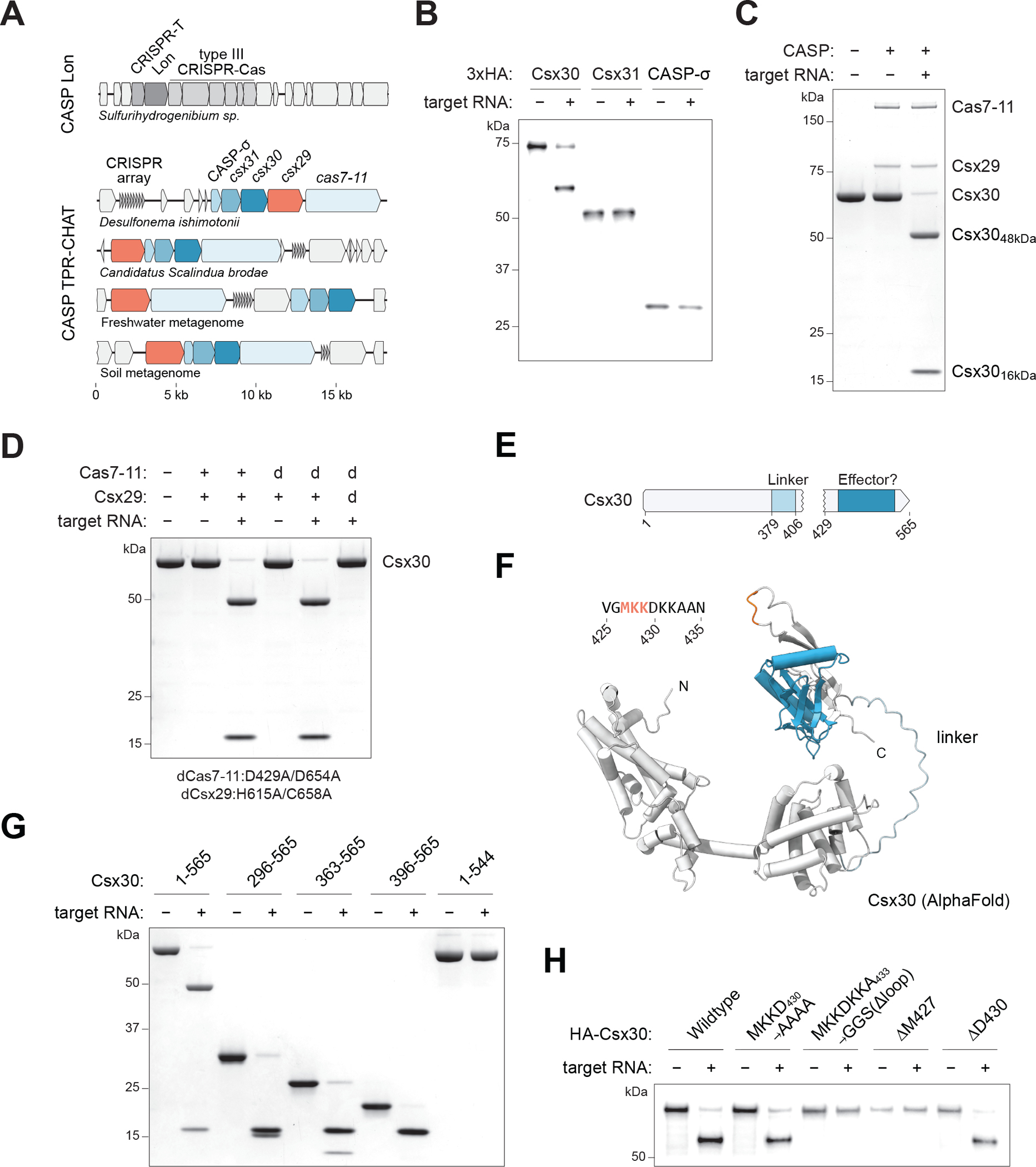
The type III-E CRISPR-associated protease Csx29 cleaves Csx30. (A) Schematic of selected CRISPR-associated protease (CASP) loci and three additional conserved genes in type III-E loci. (B) Immunoblot analysis of in vitro reactions with Cas7–11-Csx29 and HA-tagged Csx30, Csx31, and CASP-σ produced by cell-free transcription-translation. (C) A Cas7–11-Csx29-crRNA complex cleaves Csx30 protein in response to target RNA. (D) Csx30 cleavage requires target RNA and the Csx29 protease catalytic residues, but not the catalytic residues of Cas7–11. (E) Schematic of Csx30 highlighting the cleavage site (aa 427–429), linker (aa 377–406), and a potential effector domain annotated from HHpred (aa 452–545). (F) AlphaFold2 prediction of Csx30 (G) Analysis of dCas7–11-Csx29 proteolytic activity on truncated Csx30 proteins. (H) Immunoblot analysis of HA-tagged Csx30 mutants produced by cell free transcription-translation. Panels C, D, and G are SDS-PAGE gels stained with Coomassie.

The reported cleavage of CRISPR-T by the neighboring Lon protease (11) inspired us to look more closely at type III-E loci for potential substrates. In addition to the associated Csx29 protease, these loci frequently contain three additional genes (*csx30*, *csx31*, and a predicted sigma factor (3), hereafter CASP-σ) that we hypothesized were prime candidates (Fig. 1A, fig. S1). Starting from a system found in *D. ishimotonii* (DiCASP) (12), we purified a stable Cas7–11-Csx29-crRNA complex (as previously reported for *Candidatus S. brodae (13)*) (fig. S2A) and performed in vitro reactions by adding the proteins expressed from the three upstream genes in the presence or absence of a target RNA complementary to the crRNA. We identified that the largest protein, Csx30, is specifically cleaved in response to a target RNA (Fig. 1, B and C). Moreover, in vitro reactions yielded two precise protein products indicating a single cleavage event within Csx30 as opposed to processive protein degradation.

We determined the requirements of Csx30 cleavage and found that while mutating the catalytic residues of the Csx29 protease (H615A/C658A) abolished activity, disrupting the catalytic sites of the Cas7–11 endonuclease (D429A/D654A) (12) did not (Fig. 1D, and fig. S2B). This result indicates that target RNA binding alone is sufficient for Csx29 activation, and that RNA cleavage is dispensable. In vitro characterization revealed that DiCASP is a highly active ATP-independent protease cleaving 100-fold molar excess of Csx30 substrate in minutes, with an optimal activity at 37–45°C (fig. S2, C–F). Full Csx30 cleavage activity required 22 nucleotides of complementarity between the crRNA and target RNA, and we detected low tolerance to base pair mismatches, particularly at the 5’ end of the target RNA (fig. S3).

## Characterization of Csx30 proteolytic processing

Structural prediction of the Csx30 protein revealed two domains separated by a flexible linker (Fig. 1, E and F) which we hypothesized to be the site of cleavage. However, mass spectrometry analysis (and the estimated 48 kDa and 16 kDa gel products) indicate that Csx30 is cleaved further downstream between residues 427 and 429 (fig. S4), placing the cleavage site within a small flexible loop (residues 423–437) in the C-terminal domain of the structural model. By generating truncation mutations of Csx30, we determined that the N-terminal domain is dispensable for processing by Cas7–11-Csx29 as Csx30 fragments containing residues 396–565 were efficiently cleaved in vitro (Fig. 1G, and fig. S5). By contrast, we observed that Csx30 C-terminal residues are strictly required and that even a twenty amino acid truncation (Csx30_1–544_) abolished cleavage activity (Fig. 1G).

Mutational analysis by alanine substitutions revealed no Csx30 residues that are essential for cleavage, although some reduced the efficiency (Fig. 1H, and fig. S6). Instead, the size of the cleaved loop appears important for processing. We observed that truncating the loop by four residues, or deleting M427 alone, prevented Csx30 cleavage, while the deletion of D430 had no effect (Fig. 1H). Using an uncleavable Csx30_Δloop_ mutant as bait, we pulled down Cas7–11-Csx29 complex both in the presence and absence of target RNA, suggesting that Csx30 binding to Cas7–11-Csx29 is not regulated by target RNA recognition or activation of the protease (fig. S7). In contrast, we did not detect Cas7–11-Csx29 binding using a truncated Csx30_1–544_ mutant, revealing that an intact C-terminal domain is required for substrate binding (fig. S7).

## Allosteric activation of Csx29 upon target RNA binding

**Fig. 2.**
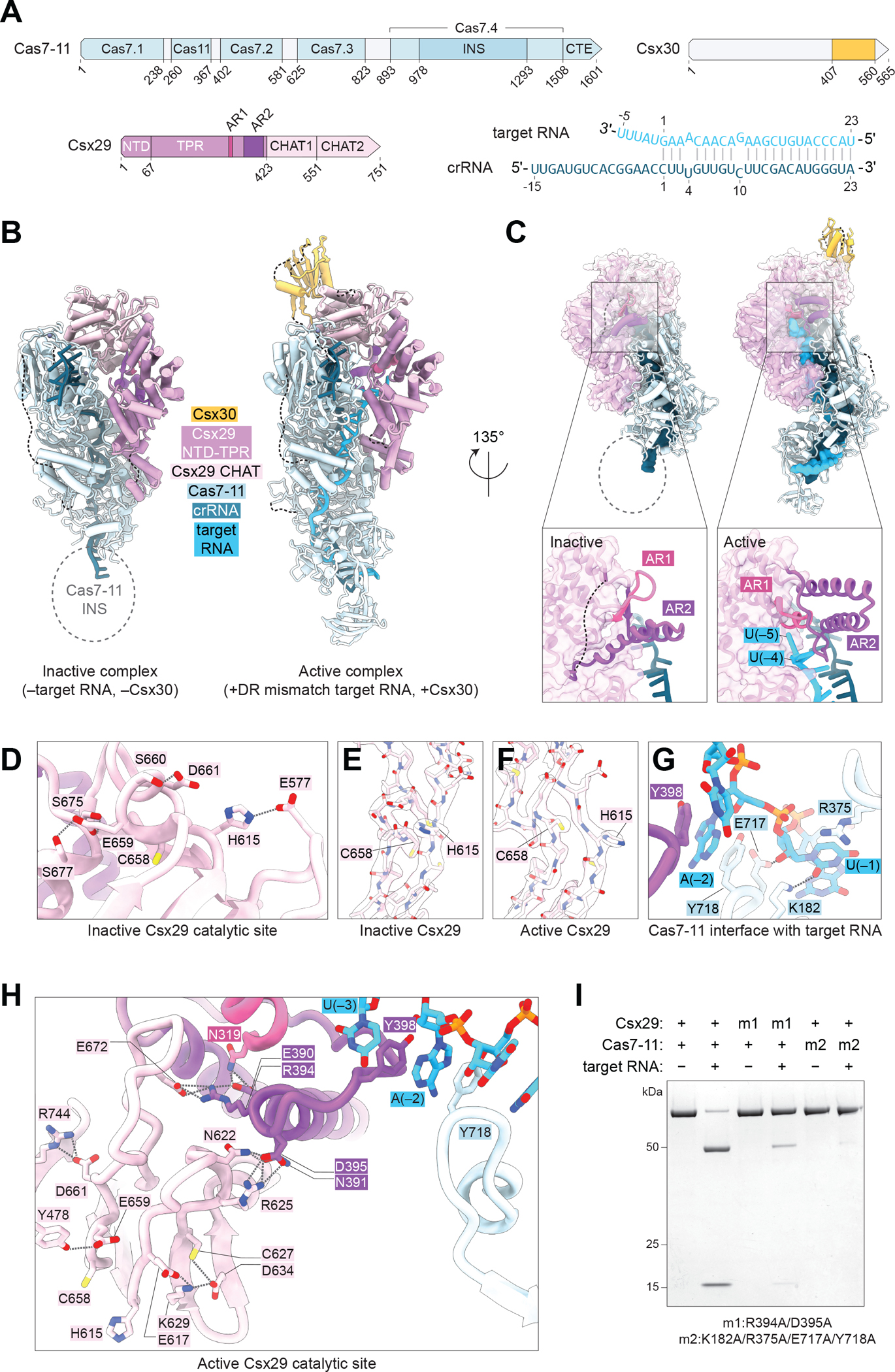
Allosteric activation of Csx29 upon RNA binding. (**A**) Schematic of Cas7–11, Csx29, and Csx30 proteins domains, and the crRNA and target RNA used in structural studies. (**B**) Structures of the inactive (Cas7–11-Csx29-crRNA) and active (Cas7–11-Csx29-crRNA-target RNA-Csx30) CASP complexes. (**C**) Structural organization of the Csx29 AR in inactive and active CASP complexes. (**D**) Electrostatic and hydrogen bonded network within the Csx29 catalytic site in the inactive state. (**E** and **F**) Catalytic H615 and C658 residues in inactive and active Csx29 shown with EM density. (**G**) Contacts between Cas7–11 and the DR-mismatched portion of the target RNA in the active state. (**H**) Electrostatic and hydrogen bonded network extending from the AR to the Csx29 catalytic site in the active state. (**I**) Mutations disrupting allosteric activation residues impair Csx30 cleavage by Cas7–11-Csx29. SDS-PAGE gel stained with Coomassie.

To gain insight into the activation mechanism of Cas7–11-Csx29 and substrate recognition of Csx30 we solved single particle cryo-electron microscopy (cryo-EM) structures of Csx30_Δloop_ bound to Cas7–11-Csx29 with target RNA, and an inactive complex of Cas7–11-Csx29 alone, at 2.5-Å and 3.0-Å resolution respectively (Fig. 2, A–C, figs. S8 to S10, and table S1). The overall architecture of Cas7–11 in both complexes resembles the reported DiCas7–11 structure (16), in which the Cas7.1-Cas7.4 domains organize into a filament around the crRNA core with Cas11 at the midpoint. The insertion (INS) domain within Cas7.4 was visible only in the active state (Fig. 2, B and C). Csx29 consists of a three-helix bundle N-terminal domain (NTD), a TPR domain with eight repeats, and a protease region containing a pseudo-caspase (CHAT1) and active-caspase (CHAT2) domain that resembles separases (17, 18). In both complexes, Cas7.2-Cas7.4 interface with the NTD, TPR and CHAT1 domains of Csx29. Although the overall organization of Cas7–11 remains the same upon Csx29 binding, linker L2 and the Cas7.4 zinc-finger loop undergo structural changes which look similar in both active and inactive states (fig. S11).

In the inactive state, the catalytic residues of CHAT2 are improperly positioned; C658 is turned downward away from the catalytic H615, and the catalytic histidine is instead positioned toward D661 (fig. S12). However, they are repositioned upon target RNA binding to resemble the geometry of active caspases (Fig. 2, D–F, fig. S12, and fig. S13). As CHAT2 makes no direct contact with Cas7–11 or target RNA, we hypothesized that conformational changes likely occur in other regions of Csx29 and transduce an allosteric signal to the catalytic core. By comparing the inactive and active complexes we observed a major structural change within the eighth repeat of the TPR domain, which we term the activation region (AR). The AR is bipartite, composed of AR1 (aa 313–325) and AR2 (aa 356–411), which stack with each other in the inactive state (Fig. 2C). In the active complex, AR1 senses the 3’ end of target RNA (position −4 and −5) through base stacking interactions and pushes the AR2 helices away, preventing a steric clash (Fig. 2C).

The target RNA in our active complex is non-complementary to the direct repeat (DR) and the structure reveals that this is an important feature. In this state, the 3’ portion of the target RNA is separated from the crRNA, and it makes a sharp kink at position −2, enabling it to traverse the TPR domain of Csx29 and reach AR1 (fig. S14A). This observation suggests that a DR-matched RNA might not activate Csx29 as it could stay hybridized with the crRNA at position −2 and beyond. Supporting this model, a target RNA fully matching the DR strongly reduced Csx30 cleavage (fig. S14, B and C). Mismatches at position −1 and −2 alone were only able to partially activate Csx29, and mismatches at −1 to −4 were required to restore full Csx30 cleavage (fig. S14C). Eliminating base pairing between the DR and the target RNA is therefore crucial for CASP activation and highlights the importance of the AR1-target RNA interaction. Of note, non-complementarity between the DR and target RNA also plays an important role in type III-A and III-B CRISPR systems to suppress the response against host derived transcripts (19, 20), and thus is a generalized component of signal transduction in type III systems.

In addition to target RNA sensing by Csx29 AR1, we identified contacts between Cas7–11 and target RNA at the DR-mismatched site. In addition to Y718 which base-stacks with the nucleotide at position −2, we identified K182, R375, and E717 contacting the nucleotide at position −1 (Fig. 2G, and fig. S13). To better understand CASP activation and the AR-induced signal transduction, we examined downstream allosteric events in Csx29. In the active complex, the kinked target RNA site at position −2 is stabilized by base stacking interactions, provided by both Cas7–11-Y718 and Csx29-Y398 within AR2. Adjacent residues at the tip of the AR2 helix, E390, N391, R394, and D395, initiate a network of electrostatic and hydrogen bonded contacts extending all the way to the CHAT2 active site (Fig. 2H, and fig. S13). Prominent salt bridges formed between R394-E672 and D395-R625 help position the loop containing the catalytic C658, and the strand containing the catalytic H615, respectively. Further down, the active site H615 is positioned by E617 contacts, whereas the active site C658 is kept in place by E659-Y478 and D661-R744. In the inactive state, these same residues positioning C658 in the active complex make entirely different contacts, E659 forms hydrogen bonds with S675 and S677, and D661 instead bonds with S660 (Fig. 2, D and H, and fig. S13). We note the similarity of this mechanism to eukaryotic caspases which are also thought to be regulated by the conformation of the L4 loop containing their catalytic cysteine (21). Together, these structures reveal an allosteric cascade initiated by the 3’ end of DR-mismatched target RNA, triggering the AR within the Csx29 TPR domain, and transducing structural changes to the Csx29 CHAT2 domain to coordinate active site residues.

To test this model, we made mutations in the allosteric network. A Csx29-R394A/D395A double mutant within AR2 formed a stable Cas7–11-Csx29 complex, but Csx30 cleavage was significantly impaired (Fig. 2I, and fig. S14D). Further down the allosteric cascade, mutating Csx29-E659 and D661 in the vicinity of the catalytic C658 likely disrupted Csx29 folding and we were unable to purify a Cas7–11-Csx29 complex. Finally, we tested the importance of contacts between Cas7–11 and target RNA at the DR-mismatched site. Mutating Cas7–11-K182, E717, R375, and Y718 into alanines did not impair Cas7–11-Csx29 complex assembly, however, strongly reduced CASP activation upon target RNA binding (Fig. 2I, and fig. S14D). Thus, target RNA stabilization by Cas7–11 on the DR-mismatched end is also critical for protease activation.

## Csx30 recognition by Cas7–11-Csx29

**Fig. 3.**
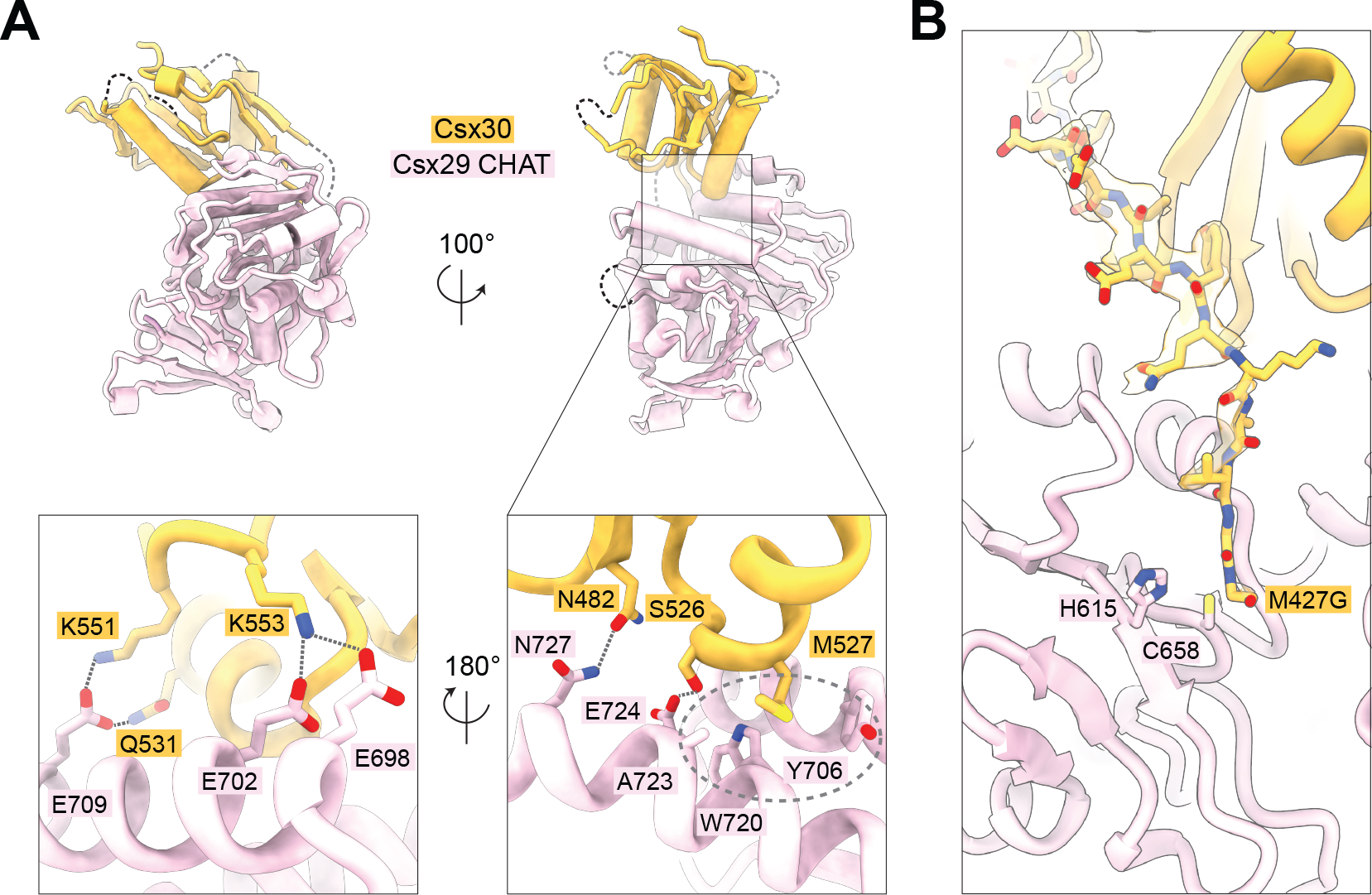
Csx30 substrate recognition by Csx29. (**A**) Csx29-Csx30 interface in the active CASP structure. Electrostatic interactions and hydrogen bonds are drawn as dashed lines, and the hydrophobic pocket as a dashed oval. (**B**) Close-up of the Csx29-Csx30 interface near the catalytic H615 and C658 residues.

In addition to revealing insight into CASP activation, our active complex also provides structural details regarding the interaction with Csx30. Despite using a full-length Csx30_Δloop_ mutant for complex assembly, only a small portion (aa 407–560) is visible in our structure (Fig. 3A, fig. S15A), and the remaining residues must therefore be flexible with respect to Cas7–11-Csx29. This region mirrors the minimal substrate we identified via truncation experiments and confirms that recognition of Csx30 is mediated through its C-terminal domain. In our structure, Csx30 is bound only to the Csx29 CHAT2 domain and does not interact with Cas7–11.

There is striking charge complementarity at the Csx29-Csx30 interface, and substrate recognition is likely electrostatically driven through the negatively charged surface of Csx29 and positively charged surface of Csx30 (fig. S15B). Detailed analysis of the interface reveals that Csx30 polar and positively charged residues (N482, S526, Q531, K551, and K553) make contact with the Csx29 CHAT2 domain (Fig. 3A, and fig. S16). In addition, Csx30-M527 is enclosed in a tight hydrophobic pocket lined with Csx29’s Y706, W720, and A723. The major determinant of Csx30 engagement is likely a cumulative effect of these interactions, as mutating individual regions of the Csx29-Csx30 interface did not significantly affect Csx30 cleavage (fig. S15C). Consistent with our ability to pulldown a Cas7–11-Csx29-Csx30_Δloop_ complex in the presence and absence of target RNA (fig. S7), the interfacing residues of Csx29 adopt a similar organization in both the active and inactive complexes, and therefore we conclude that Csx30 binding is not allosterically regulated.

We also examined the position of the Csx30 cleavage site within the active complex. One limitation of our structure is that the cleavage loop is mutated (and slightly shortened), and thus, we cannot observe substrate engagement in the active site in detail. As the loop is also flexible, it is not well resolved, but its density places it near the active site of Csx29 positioning it for cleavage (Fig. 3B).

## Csx30 binds and inhibits the transcription factor CASP-σ

We next sought to explore the biological function of Csx30 and understand how cleavage might regulate its activity. As the Cas7–11 effector alone provides defense against phage (12), we reasoned that additional functions of DiCASP would similarly be involved in the immune response. One possibility is that processed Csx30 fragments, Csx30-N (residues 1–428) or Csx30-C (residues 429–565), promote cell death or an abortive infection response to prevent phage propagation. However, we did not observe defense against three tested phage (fig. S17A). Homology searches revealed a match of Csx30-C to a peptidoglycan N-acetylglucosamine deacetylase (HHpred probability: 92.85%, e-value: 0.56), but we did not detect modification of peptidoglycan or its components with cleaved Csx30 in vitro (fig. S17B). Overexpression of Csx30 fragments was not toxic in *E. coli*, and we only observed a slight growth defect in cells expressing full-length Csx30, which was temperature dependent and suppressed by the addition of Csx31 and CASP-σ (fig. S18).

**Fig. 4.**
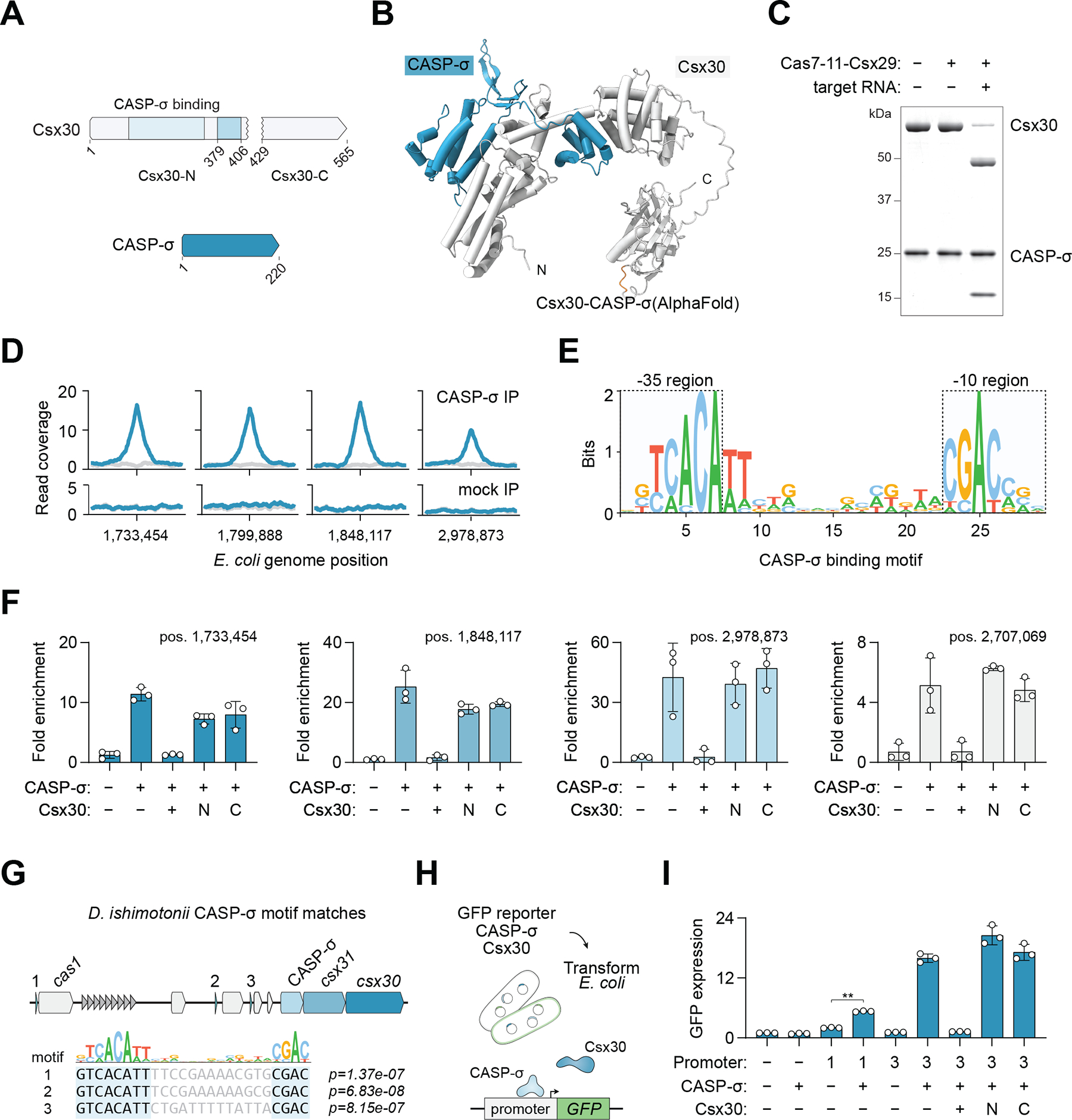
Csx30 binds and inhibits the transcription factor CASP-σ. (**A**) Schematic of Csx30 and CASP-σ proteins. (**B**) AlphaFold2 prediction of a Csx30-CASP-σ interaction. (**C**) Purification of a Csx30-CASP-σ complex that is cleaved by dCas7–11-Csx29. SDS-PAGE gel stained with Coomassie. (**D**) Representative CASP-σ ChIP-seq peaks in *E. coli* with a 1 kb window, input coverage shown in gray. (**E**) Identification of a CASP-σ binding motif from ChIP-seq peaks. (**F**) Enrichment of CASP-σ at four *E. coli* peaks by ChIP-qPCR. n = 3 replicates. (**G**) Predicted CASP-σ targets in the *D. ishimotonii* CASP locus. (**H**) Schematic of a CASP-σ transcriptional reporter assay. (**I**) CASP-σ-mediated transcriptional activity in *E. coli.* GFP expression was normalized to cells with a scrambled promoter sequence. n = 3 replicates. ** denotes p < 0.01, Student’s t-test. Error bars represent standard deviation from the mean in all panels.

We next turned to the other proteins encoded in the locus to gain insight into Csx30 function. We predicted a strong binding interaction between the N-terminal domain of Csx30 and CASP-σ, which strongly resembles an extracytoplasmic function (ECF) sigma factor (3) (HHpred probability 100%, e-value 3.4e-31) (Fig. 4, A and B, and fig. S19). Sigma factors are transcription initiation proteins that bind DNA and recruit the RNA polymerase catalytic core to specific promoters (22), hinting that Csx30 might be involved in regulating a transcriptional response. Consistent with our computational prediction, purification of CASP-σ in the presence of Csx30 yielded a Csx30-CASP-σ complex, in which Csx30 could still be cleaved by Cas7–11-Csx29 (Fig. 4C). Csx30-N was sufficient for the interaction with CASP-σ, although at considerably lower yield (fig. S20).

Although *D. ishimotonii* CASP-σ is unlikely to regulate its target genes heterologously in *E. coli,* we reasoned that the identification of putative CASP-σ binding sites might yield insight into its preferred sequence motif and function in the natural host. We performed ChIP-seq in *E. coli* with HA-tagged CASP-σ and identified 13 high confidence peaks compared to input and mock IP controls (Fig. 4D, and fig. S21A). Motif analysis of ChIP-seq peaks yielded a clear hit (Fig. 4E, and fig. S21B), which was similar to a de novo predicted motif (fig. S21C) (23).

Sigma factors are frequently regulated by inhibitors (anti-sigma factors), and there are examples in bacteria in which a protease cleaves an anti-sigma factor to activate a transcriptional stress response including the anti-sigma factors RseA in *E. coli (24)* and RsiW in *B. subtilis (25)*. In *E. coli*, the DegS protease senses cell envelope stress and cleaves a transmembrane segment of RseA (26), resulting in the eventual release of the sequestered sigma factor RpoE. Based on our structural model, we predict that the Csx30-CASP-σ interaction would block CASP-σ DNA binding based on steric clashes to sigma factor-bound DNA in experimental structures (27) (fig. S22). To test whether Csx30 inhibits CASP-σ, we repeated ChIP experiments in *E. coli* co-expressing Csx30 and found that CASP-σ DNA binding was blocked at all four tested loci (Fig. 4F). This inhibition was dependent on full-length Csx30 as both Csx30-N and Csx30-C fragments were unable to antagonize CASP-σ binding (Fig. 4F). Together our results suggest that Csx30 is an inhibitor of CASP-σ, and that processing by Cas7–11-Csx29 alleviates this inhibition.

## Csx30 processing regulates CASP-σ transcriptional activity

We next sought to identify potential CASP-σ targets in the natural host *D. ishimotonii*. As many ECF sigma factors autoregulate their own expression (28), we first searched the DiCASP locus. We identified three strong matches in the promoters of *cas1* and two genes of unknown function (Fig. 4G, and table S2), indicating that CASP-σ likely coordinates additional defense functions including CRISPR spacer acquisition. Genome-wide searches for motifs in *D. ishimotonii* promoter regions yielded several candidates although only one site, upstream of the *nhaA* gene, was below a q-value of 0.6 (table S3 and S4). To test these predictions, we constructed transcriptional reporters by placing putative CASP-σ promoters upstream of green fluorescent protein (GFP) and measured the resulting fluorescence in *E. coli* (Fig. 4H, and fig. S23, A and B). We observed GFP expression with both tested promoters compared to a random DNA control and found that fluorescence was fully dependent on CASP-σ expression (Fig. 4I). Consistent with our previous results, co-expression of full-length Csx30 was able to completely inhibit CASP-σ-mediated GFP expression whereas processed Csx30 fragments had no effect (Fig. 4I). Supporting a role in the immune response, we could computationally identify one of the two unknown ORFs, a predicted membrane protein, in other CRISPR and defense loci (fig. S23C).

## RNA sensing applications with DiCASP

**Fig. 5.**
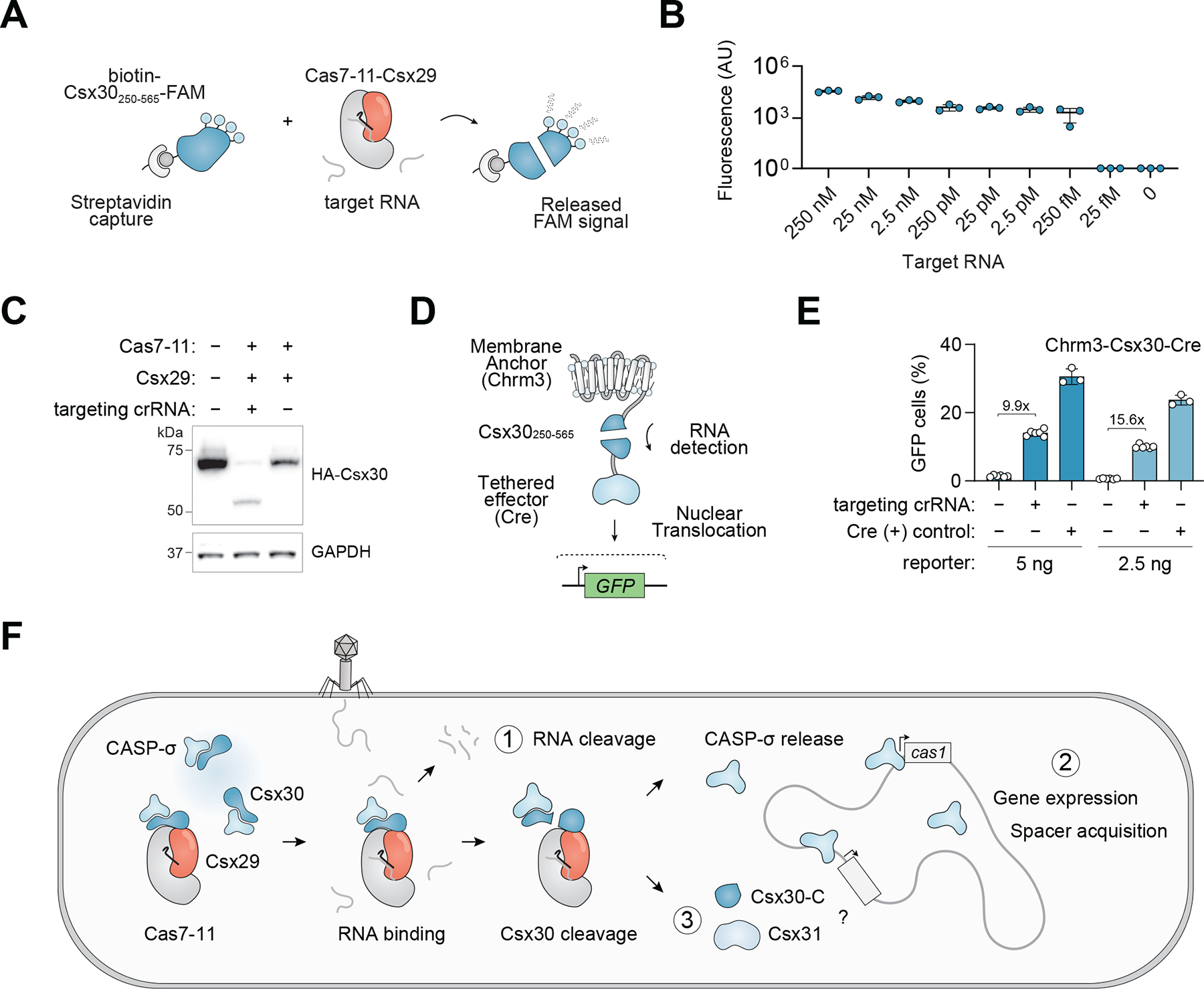
RNA sensing applications and a proposed model for CASP systems. (**A**) Schematic of in vitro RNA detection using CASP systems and fluorescent Csx30 reporters. (**B**) In vitro detection of RNA as measured by released fluorescence. n = 3 replicates. (**C**) Immunoblot analysis of HA-tagged Csx30 in HEK293T human cells transfected with DiCASP components. (**D**) Schematic of engineered proteins containing a cell membrane tether, a Csx30 linker, and an effector domain. (**E**) Flow cytometry of DiCASP activity in mouse Neuro2A loxP:GFP cells using a Chrm3-Csx30_250–565_^−^Cre reporter. n = 3–6 replicates. (**F**) Model for a three-pronged strategy of CASP systems in the defense against foreign genetic elements including Cas7–11 mediated RNA endonuclease activity, a Csx30-regulated CASP-σ transcriptional response, and a possible third arm involving Csx31. Error bars represent standard deviation from the mean in all panels.

The high proteolytic activity of Cas7–11-Csx29 in response to a target RNA enables numerous biological applications. In addition, the ability to uncouple RNA cleavage from activation of the Csx29 protease allows for non-destructive sensing of RNA. While the collateral nuclease activity of CRISPR effectors has been used to cleave nucleic acid-based reporters for diagnostic applications (29), CASP systems allow for a new modality of substrates using engineered Csx30 proteins. As a proof of concept, we generated a fluorescently labeled engineered variant of Csx30 and demonstrated its ability to detect RNA in vitro down to 250 femtomolar without nucleic acid amplification (Fig. 5, A and B, and fig. S24).

We also sought to apply DiCASP for RNA transcript sensing in live cells. To determine if DiCASP can mediate RNA-activated proteolytic cleavage in human cells, we transfected plasmids expressing Cas7–11, Csx29, crRNA, a synthetic target RNA, and Csx30 fused to an HA epitope tag into HEK293T cells. Immunoblots of cell lysate revealed processing of Csx30 that was dependent on a targeting crRNA and the catalytic residues of the Csx29 protease (Fig. 5C, and fig. S25, A and B). Testing DiCASP activity across a panel of endogenous transcripts revealed Csx30 cleavage efficiencies ranging from 2 to 20% (fig. S25, C and D).

To convert RNA sensing with DiCASP into a discrete and readily detectable signal we sought to design reporters containing effector domains that could be activated by Csx30 cleavage. We transfected plasmids encoding a fusion protein in which Cre recombinase is tethered to membrane anchors (e.g. the cholinergic receptor, muscarinic 3 (Chrm3) GPCR) via a Csx30-derived linker, sequestering Cre from the nucleus (Fig. 5D). Mouse Neuro-2A cells harboring an inactive loxP-GFP reporter cassette were transfected with DiCASP components and synthetic target RNA. Flow cytometry analysis revealed crRNA-dependent GFP expression in 10% of cells, and a 15-fold increase over non-targeting crRNA controls under optimal conditions (Fig. 5E, and fig. S25, E and F).

## Discussion

Here we demonstrate that the Csx29 protease associated with the type III-E effector Cas7–11 mediates RNA-activated endopeptidase activity and elucidate its substrate, structure, and mechanism. Although the full biological consequence of Csx30 processing in *D. ishimotonii* is unknown, our work supports a model in which Csx30 inhibits the sigma factor CASP-σ, and that proteolytic cleavage by the Csx29 protease acts to relieve this inhibition. The parallels between DiCASP and other protease-regulated anti-sigma factors, like DegS and RseA (26), reveal convergent mechanisms for modulating gene expression in response to cellular threats. The N-terminal domain of Csx30 is sufficient for binding to CASP-σ and it is therefore unclear how proteolytic cleavage within the Csx30 C-terminal domain would release CASP-σ, or why expression of Csx30-N is unable to inhibit CASP-σ. One possibility is that the processed Csx30 fragments are unstable and that the exposed termini are subject to further degradation by host proteins. Consistent with this hypothesis, immunoblots of *E. coli* cell lysates harboring HA-tagged isoforms of Csx30 revealed expression of full-length Csx30 and Csx30-C, but not Csx30-N, and that blocking the “cleaved” termini with an epitope tag increased expression (fig. S26). We note potential similarities to other protease-regulated anti-sigma factor systems; DegS cleavage of RseA is insufficient to release the sigma factor RpoE and the remaining RseA fragment is further processed by the RseP (30, 31) and ClpXP proteases (32) to liberate RpoE.

Our identification of three CASP-σ binding motifs within the CASP locus points to the positive autoregulation of defense genes, including *cas1*, which may be a mechanism to acquire new spacers during active infection and to safeguard against the acquisition of self-targeting spacers during normal growth. This result is consistent with the reported upregulation of *cas1* in *Pseudomonas aeruginosa* by the ECF sigma factor PvdS (33). The functions of the two other predicted upregulated genes in the locus are unknown, although one has strong homology to a membrane transporter component EcsC (HHpred probability 99.9, e-value 3.1e-22). Interestingly, the top motif match outside of the CASP locus is upstream of *nhaA* (table S3), a Na+/H+ antiporter known to be upregulated during phage infection (34), indicating that CASP-σ may also regulate targets elsewhere in the genome.

Together, our results suggest the subtype III-E CASP systems use a three-pronged strategy to defend against foreign genetic material: (1) targeted RNA cleavage via the RNA endonuclease Cas7–11, (2) a Csx30-CASP-σ regulated transcriptional response that leads to, amongst other possibilities, spacer acquisition, and (3) a potential third arm mediated by Csx31 and possibly Csx30-C (Fig. 5F). The clear conservation of Csx31 (fig. S1) is a strong indication of its biological importance and future work will be required to determine its role in the immune response.

We predict similar interactions between Csx30 and CASP-σ in other type III-E systems as well as putative CASP-σ binding motifs at *cas1* within the *Candidatus S. brodae* locus (fig. S27). There may also be parallels between DiCASP and the type III CRISPR-associated Lon protease (11). We note that CRISPR-T is also associated with a neighboring sigma factor and is predicted to physically interact (fig. S28). We hypothesize that cleavage of CRISPR-T could similarly trigger transcriptional changes and may reflect a common functional theme across diverse CASP families.

This work reveals an example of CRISPR systems coordinating a wider cellular response beyond nuclease activity, and we expect that the continued investigation of CRISPR-associated enzymes will uncover many interesting, and potentially useful, RNA-activated biological processes.

## Material and Methods

### Gene synthesis and cloning

The TPR-CHAT protease and *csx30*, *csx31,* and *CASP-σ* genes from *D. ishimotonii* were codon optimized for human cell expression (GenScript) and synthesized and assembled from gene fragments. Additional materials were cloned by Gibson Assembly (New England Biolabs). pDF0159 (pCMV - huDisCas7–11, Addgene # 172507), pDF0118 (TwinStrp-SUMO-DisCas7–11, Addgene #172503), and pDF0114 (pU6-crRNA, Addgene #172508) were gifts from Omar Abudayyeh & Jonathan Gootenberg.

### In vitro RNA synthesis

In vitro transcribed RNA was generated by annealing a DNA oligonucleotide containing the reverse complement of the desired RNA with a short T7 oligonucleotide. In vitro transcription reactions were performed using the HiScribe T7 High Yield RNA synthesis kit (NEB) at 37°C for 8–12 h and RNA was purified using Agencourt AMPure RNA Clean beads (Beckman Coulter).

### Cell-free transcription-translation

3xHA tagged forms of Csx30–3 were cloned into pCDNA3.1 vectors and amplified by PCR using oligos containing the T7 promoter and terminator. Cell-free transcription-translation was performed using PURExpress (New England Biolabs) in 5 μL reactions containing 2 μL buffer A, 1.5 μL buffer B, 0.25 μL of Superase RNAse Inhibitor (Invitrogen), and 50–100 ng of PCR template. Reactions were incubated for 2 h at 37°C and directly transferred to in vitro reactions.

### Protein purification

All proteins were expressed in BL21 *E. coli* (Sigma Aldrich, CMC0016). Cells were grown in Terrific Broth (TB) to mid-log phase and the temperature was lowered to 18°C. Expression was induced at OD_600_ 0.6 with 0.25 mM IPTG for 16–20 h before harvesting and freezing cells at −80°C. The gRAMP-CHAT complex was purified following co-expression of plasmids containing TwinStrep-SUMO-gRAMP and a mature crRNA, and pCDF-6xHis-CHAT. Cell paste was resuspended in lysis buffer (50 mM Tris pH 7.5, 250 mM NaCl, and 5% glycerol). Cells were lysed using a LM20 microfluidizer (Microfluidics) and cleared lysate was bound to Strep-Tactin Superflow Plus (Qiagen) using the gRAMP affinity tag. The resin was extensively washed and bound protein was eluted by cleaving the TwinStrep-SUMO tag with 10 μg Ulp1 SUMO protease overnight at 4°C. The eluted protein was bound to Ni-NTA Superflow (Qiagen) in 15 mM imidazole using the CHAT affinity tag, the resin was extensively washed with lysis buffer plus 40 mM imidazole, and the complex was eluted with 300 mM imidazole buffer. The eluted complex was diluted to 100 mM NaCl and purified on a HiTrap Heparin (Cytiva) column with a 100 mM to 1 M NaCl gradient. Fractions containing the gRAMP-CHAT complex were pooled, concentrated, and run on a Superose 6 Increase column (Cytiva) with a final storage buffer of 25 mM Tris pH 7.5, 250 mM NaCl, 10% glycerol, 1 mM DTT. All purified proteins were flash frozen in liquid nitrogen and stored at −80°C until use.

Csx30 was purified using a TwinStrep-SUMO tag and lysis buffer containing 50 mM Tris pH 7.5, 250 mM NaCl, and 5% glycerol. Following Ulp1 SUMO protease digestion and elution from Strep-Tacin beads, Csx30 protein was diluted to 100 mM NaCl and purified using a Resource Q anion exchange column (Cytiva) with a 100 mM to 1 M NaCl gradient before gel filtration chromatography on a Superose 6 Increase column (Cytiva) with a final storage buffer of 25 mM Tris pH 7.5, 250 mM NaCl, 10% glycerol, 1 mM DTT. For pulldown experiments, Csx30 protein was eluted with 5 μM desthiobiotin instead of Ulp1 SUMO protease cleavage before ion exchange chromatography to retain the TwinStrep-SUMO tag.

CASP-σ was purified using a pCDF-6xHis-Csx30 plasmid and Ni-NTA Superflow resin (Qiagen) in lysis buffer containing 50 mM Tris pH 7.5, 250 mM NaCl, 1 mM MgCl_2_, 5% glycerol and 15 mM imidazole. The resin was extensively washed with lysis buffer plus 40 mM imidazole, and CASP-σ eluted with 300 mM imidazole buffer. The Csx30-CASP-σ complex was purified in a similar way with the addition of a pUC19 plasmid containing untagged Csx30. The complex was purified using a Resource Q anion exchange column (Cytiva) following CASP-σ elution and moved to storage buffer (25 mM Tris pH 7.5, 250 mM NaCl, 10% glycerol, 1 mM DTT).

### Csx30 in vitro reactions

Typical in vitro reactions were performed in 20 μL containing 4 μL of 5x reaction buffer (100 mM HEPES pH 7.5, 500 mM NaCl, 5 mM DTT, 25% glycerol), 0.5 μL of 150 mM MgCl2, 1 μL of Csx30 substrate (2.5 uM final concentration), 2 μL of gRAMP-CHAT-crRNA complex (25 nM final concentration), and 2 μL of purified target RNA (250 nM final concentration) unless otherwise noted. Reactions were incubated at 37°C for 1 hour before the addition of Laemmli buffer. Samples were boiled for 5 minutes and run on 12-well Nupage 4–12% Bis-Tris gels (Invitrogen) and stained with Coomassie dye before imaging on a Chemi-Doc (Bio-Rad). Biochemical experiments were typically performed with two independent replicates and a representative gel image shown.

### Mass spectrometry analysis

Gel bands were excised from Coomassie stained SDS-PAGE gels following analysis of in vitro reactions and analyzed by the Whitehead Proteomics Core Facility using trypsin and chymotrypsin digests.

### CASP complex formation for cryo-EM

Protein purification for the inactive CASP complex was performed as described above with the following modifications: (1) A pETDuet-1 derived plasmid containing His14-TwinStrep- bdSUMO-Cas7–11 with D429A/D654A mutations and a mature crRNA, and a pCDF-6xHis-Csx29 plasmid were used for co-expression; (2) bdSENP protease was used to cleave the His14-TwinStrep-bdSUMO tag from the Cas7–11-crRNA-Csx29 complex on Strep-Tactin resin; (3) after performing Heparin column purification, the complex was dialysed against a final storage buffer containing 20 mM Tris pH 8.0, 250 mM NaCl, 2.5% glycerol, concentrated, flash frozen in liquid nitrogen and stored at −80°C until use. For the active CASP complex, purification was carried out similarly, and Csx30_Δloop_ retaining the TwinStrep-SUMO tag was purified separately. After Heparin column purification, the Cas7–11-crRNA-Csx29 complex was mixed with target RNA and TwinStrep-SUMO-Csx30_Δloop_ in 1:10:10 molar ratio, in a buffer condition containing 20 mM Tris pH 8.0, 100 mM NaCl, 5% glycerol, and incubated at 37°C for 30 min. The mixture was then bound to Strep-Tactin resin, and the TwinStrep-SUMO tag was cleaved with SUMO protease Ulp1 to elute the Cas7–11-crRNA-target RNA-Csx29-Csx30 complex. The complex was run on a Superose 6 Increase column (Cytiva) with a final storage buffer of 20 mM Tris pH 7.5, 100 mM NaCl, 1% glycerol, concentrated, flash frozen in liquid nitrogen and stored at −80°C until use.

### Cryo-EM sample preparation

For cryo-EM, the inactive CASP complex was diluted to 1 μM in a final buffer containing 20 mM Tris pH 7.5, 100 mM NaCl, 0.5% glycerol, and the active CASP complex was used at 1.6 μM in its final storage buffer. Quantifoil R1.2/1.3 300 mesh Cu holey carbon grids (Quantifoil, Germany) were glow-discharged (EMS 100, ElectronMicroscopy Sciences) at 25 mA for 1 min. 3 μl of each sample was applied to glow-discharged grids, blotted for 5 s using Standard Vitrobot Filter Paper (Ted Pella), and plunge-frozen in liquid ethane using a Vitrobot Mark IV (Thermo Fisher Scientific) at 4°C and 100% humidity.

### Cryo-EM data collection

All data were collected at liquid nitrogen temperature on a Titan Krios G3i microscope (Thermo Scientific), equipped with a K3 direct detector (Gatan), operated at an accelerating voltage of 300 kV, and an energy filter with slit width of 20 eV. Movies were recorded in super-resolution mode with twofold binning at 130,000× magnification giving a physical pixel size of 0.6632 Å, with a 0.5–2.0 μm defocus range, at an electron exposure rate of 25.5 e−/pix/s for 0.69 s, fractionated into 30 frames, resulting in an accumulated fluence of 40 e−/Å2 per micrograph. 16,553 movies for the inactive complex, and 10,963 movies for the active complex were collected.

### Cryo-EM data processing

All cryo-EM data were processed using RELION-4.0 (36), compiled and configured by SBGRid (37). Movies were corrected for motion using the RELION implementation of MotionCor2, with 5-by-5 patches and dose-weighting, and Contrast Transfer Function (CTF) parameters were estimated using CTFFIND-4.1 (38). For both datasets, particle picking was carried out using the Topaz general model (39). All reported resolutions use the gold-standard Fourier shell correlation with a cutoff of 0.143.

For the inactive complex, 877,928 particles were extracted from 16,553 micrographs, and downscaled twofold. Analysis of these particles by 2D classification (100 classes, tau_fudge = 2, 220 Å mask diameter) revealed a mixture of dimers and monomers (fig. S7), and a monomeric reference model generated using RELION on a preliminary dataset collected on a Talos Arctica microscope was used for reconstruction. After cleaning poor quality particles by 3D classification (4 classes, tau_fudge = 4, 30 Å resolution reference, 25 iterations), remaining particles were subject to CTF refinement and Bayesian polishing, and one more round of 3D classification (4 classes, tau_fudge = 4, 15 Å resolution reference, 25 iterations, soft mask with 3 pixel hard edge, 8 pixel soft edge), and refinement, producing a reconstruction from 374,026 particles at 3.2-Å resolution. Since the peripheral regions of the complex, as well as Csx29 NTD, and the NTD-proximal parts within the TPR domain were flexible, focused refinement was performed to improve the EM density in those regions. A mask encompassing Csx29 NTD, as well as the well-ordered core region of Cas7–11, including crRNA was generated, and 3D classification without alignment (4 classes, tau_fudge = 100, 6 Å resolution reference, 30 iterations), showed that 71% of particles did not have strong density within this masked region. After removing these particles, the remaining particles were focus-refined by performing local angular searches starting at 0.9 degree sampling, first using the classification mask, and then using a mask encompassing the entirety of Cas7–11 and Csx29 NTD, producing a reconstruction at 3.0-Å resolution. Focused refinement efforts on the Cas7–11 INS domain were not successful. To improve the density for Csx29 TPR and CHAT, a mask encompassing only these two domains was produced, and 3D classification without alignment (4 classes, tau_fudge = 100, 6 Å resolution reference, 30 iterations), showed that 76% of particles did not have strong density within the masked region. After removing these particles, the remaining particles were focus-refined by performing local angular searches starting at 0.9 degree sampling, and using the classification mask, producing a reconstruction at 3.2-Å resolution.

For the active complex, 2,143,080 particles were extracted from 10,963 micrographs, and downscaled twofold. Unlike the inactive complex, 2D classification analysis (200 classes, tau_fudge = 2, 220 Å mask diameter) revealed only monomers (fig. S8). After cleaning poor quality particles by 3D classification (4 classes, tau_fudge = 4, 30 Å resolution reference, 25 iterations), remaining particles were subject to CTF refinement and Bayesian polishing, and one more round of 3D classification (4 classes, tau_fudge = 100, 10 Å resolution reference, 30 iterations, soft mask with 3 pixel hard edge, 8 pixel soft edge), and refinement, producing a reconstruction from 187,426 particles at 2.4-Å resolution. Similar to the inactive complex, the peripheral regions of the overall refined active complex had weaker EM density compared to the core, and the density for the Cas7–11 INS domain, and Csx30 was mostly blurred, so focused refinement was performed to improve the map in those regions. A mask encompassing only the Cas7–11 INS domain was generated, and 3D classification without alignment (4 classes, tau_fudge = 200, 10 Å resolution reference, 30 iterations), showed that 65% of particles did not have strong density within this masked region. After removing these particles, the remaining particles were focus-refined by performing local angular searches starting at 0.5 degree sampling, using the classification mask, producing a reconstruction at 2.8-Å resolution. The same particles were further focus-refined afterwards, by performing local angular searches starting at 0.9 degree sampling, and using a mask encompassing the entirety of Cas7–11, producing a reconstruction at 2.5-Å resolution. To improve the density for Csx29 and Csx30, a mask encompassing only the Csx29 CHAT domain, and Csx30 was produced, and 3D classification without alignment (4 classes, tau_fudge = 100, 10 Å resolution reference, 30 iterations), showed that 65% of particles did not have strong density within the masked region. After removing these particles, the remaining particles were focus-refined by performing local angular searches starting at 0.5 degree sampling, using the classification mask, producing a reconstruction at 2.7-Å resolution. The same particles were further focus-refined afterwards, by performing local angular searches starting at 0.5 degree sampling, and using a mask encompassing the entirety of Csx29 and Csx30, producing a reconstruction at 2.6-Å resolution.

### Model building

Initial protein models were generated using AlphaFold2 (40) and fit into the cryo-EM maps, and then manually edited using Coot (41), while RNA molecules were entirely *de novo* built in Coot. All models were further refined in ISOLDE (42). Coordinates were refined in real space using PHENIX (43), performing one macrocycle of global minimization and atomic displacement parameter (ADP) refinement and skipping local grid searches. Statistical validation for the final models was performed using PHENIX, RNA geometry was checked using the MolProbity server (44), and 3D-FSC sphericity values were calculated using 3D-FSC server (45).

### Phage plaque assays

*E. coli* strains containing CASP expression plasmids were grown overnight at 37°C in LB with the appropriate antibiotic. 500 μL of each culture was diluted in 10 ml of molten top agar (10 g/L tryptone, 5 g/L yeast extract, 10 g/L NaCl, 7 g/L agar) and poured onto LB plates containing the appropriate antibiotic. Phage were diluted ten-fold in phosphate-buffered saline (PBS) and spotted onto dried top agar plates. Plates were incubated overnight at 37°C and imaged in a dark room with a white backlight.

### Thin layer chromatography

Uridine 5′-diphospho-N-acetylglucosamine (UDP-GlcNAc, Sigma Aldrich U4375), N-acetylemuramic acid (MurNAc, Sigma Aldrich A3007), and peptidoglycan from *Bacillus subtilis* (Sigma Aldrich, 69554) were resuspended in dimethyl sulfoxide at 10 mg/mL. Full-length or cleaved Csx30 protein was added and the reactions incubated at 37°C for 2 hours in the presence of 1 mM MgCl_2_, 1 mM ZnCl_2_, and 5 mM DTT. Oligosaccharides were separated by thin layer chromatography on silica gel 60 F254 LuxPlates (Millipore Sigma) in 30% propanol for 1 hour, and charred with 30% ammonium bisulfate at 150°C for 15 min for visualization. UDP-GlcNAc was visualized under 254 nm UV light.

### E. coli growth experiments

Stbl3 (Thermo Fisher Scientific, C737303) and TOP10 cells (Thermo Fisher Scientific, C404010) were transformed with pUC19 and pBAD derived plasmids respectively. Cells were grown overnight in LB with the appropriate antibiotic to stationary phase. For liquid culture experiments, 3 μL was used to inoculate 150 μL cultures in clear 96-well plates. Plates were sealed with clear optical film and two holes were punched for aeration using a 28 gauge needle. Plates were incubated in a Synergy Neo2 plate reader (BioTek) at the indicated temperature with constant orbital shaking and the optical density at 600 nm read every 5 minutes. Plate-based growth assays were performed by normalizing the input density of overnight cultures and performing 10-fold dilutions. 5 μL of each dilution was dropped onto agar plates and grown at the indicated temperature for 16 hours. Plates were imaged using a Chemi-Doc (Bio-Rad).

### Csx30 labeling and in vitro diagnostics

To prevent labeling of Csx30-N amine side chains, we mutated eight lysine residues to arginine, and four lysines within the cleavage loop to alanine. Mutated and truncated Csx30 was purified as previously described except with HEPES buffer in all steps instead of Tris. Csx30 was biotinylated in vitro using the BirA biotin ligase (Avidity). Csx30 was incubated with NHS-Fluorescein (Thermo Fisher Scientific, #46409) on ice for 1 h before quenching with 200 mM Tris pH 7.5. Labeled Csx30 was purified using a Resource Q anion exchange column as before. Purified biotin-Csx30-FAM substrate was bound to MyOne Streptavidin T1 dynabeads (Thermo Fisher Scientific) in phosphate buffered saline (PBS) for 30 min at room temperature. The beads were washed 10 times with PBS supplemented with 0.1% bovine serum albumin and resuspended in PBS. In vitro reactions were performed as before and Dyneabeads were removed from the reaction using a magnetic stand. The supernatant, containing cleaved Csx30C, was transferred to 96-well plates and fluorescence measured using a Synergy Neo2 plate reader (BioTek) and subtracting the background signal from a well with no target RNA.

### ChIP-seq library preparation

BL21 cells (Sigma Aldrich, CMC0016) expressing HA-CASP-σ were grown in 25 mL cultures in LB to mid-log phase and induced with 0.25 mM IPTG for 3 h at 37°C. Formaldehyde was added (1% final concentration) and cells incubated for 5 min before quenching with 275 mM glycine pH at 4°C for 20 min. Cells were washed in ice-cold Tris buffer saline and stored at −80°C until processing. Pellets were resuspended in 500 μL lysis buffer (10 mM Tris pH 8.0, 20% sucrose, 50 mM NaCl, 10 mM EDTA, 10 mg/mL lysozyme) and sonicated with a microtip probe (QSonica) to shear DNA. Lysates were spun for 15 min at 4°C at 21,000 g and 2 mL of immunoprecipitation buffer was added (50 mM HEPES pH 7.5, 150 mM NaCl, 1 mM EDTA, 1% Triton X-100, 0.1% Sodium deoxycholate) with a sample taken as an input control.

HA-CASP-σ immunoprecipitation was performed by adding 50 μL of washed Pierce Anti-HA Magnetic Beads (Thermo Fisher Scientific) and incubating at 4°C for 4 hours. Beads were washed 3 times with immunoprecipitation buffer, 3 times with wash buffer (10 mM Tris pH 8, 250 mM LiCl, 1 mM EDTA, 0.5% NP-40, 0.5% Sodium deoxycholate), and 2 times with TE (10 mM Tris pH 8, 1 mM EDTA). DNA was eluted with 100 μL TE supplemented with 1% SDS and a 65°C incubation for 10 min. 340 μL of TE with 40 μg RNAse A was added and samples incubated at 37°C for 2 hours. Formaldehyde cross-links were reversed by overnight incubation at 65°C and DNA was purified using Qiagen PCR Purification columns. DNA was sequenced using the NEBNext Ultra II DNA Library Prep Kit for Illumina (New England Biolabs) and an Illumina MiSeq.

### ChIP-seq analysis

Reads were mapped as .fastq files to *E. coli* K12 MG1655 (NC_000913.3) using http://browsergenome.org (46) with mapping parameters: no read filter, forward mapping start = 0 bp, forward mapping length = 25 bop, reverse mapping length = 15 bp, max forward/reverse span = 1000 bp, discard ambiguous hits. Mapped reads were exported as .SAM files and imported into Geneious (v2022.1.1) where coverage tables were extracted. Reads mapping to LacI (NC_000913.3:366000–368000) were filtered out due to the presence of the LacI on a plasmid used for ChIP. Remaining reads were normalized to the median per base coverage as there is a long right tail in the reads per base distribution. Putative peaks were identified as regions where the normalized coverage was greater than 4 in the CASP-σ IP samples and less than 3 in the control IP samples using Python. Peaks were then visually examined to ensure that their shape matched the expected triangular structure of a localized ChIP-seq peak. The 60 bps centered at the max coverage position of the 13 remaining peaks were aggregated and fed into MEME (https://meme-suite.org/meme/tools/meme, version 5.4.1) (47), producing a single strong hit based on 12 of the 13 loci. A putative binding site was identified manually in the remaining sequence (NC_000913.3:3880776–3880799) and logos were generated from all 13 loci using LogoMaker (48) in a Jupyter Notebook. Scripts for analysis and generating figures and tables can be found in the Zenodo repository.

### ChIP-qPCR

BL21 cells (Sigma Aldrich, CMC0016) co-transformed with plasmids expressing HA-CASP-σ and Csx30 isoforms were grown, formaldehyde fixed, and frozen as previously described for ChIP-seq analysis. Cell pellets were resuspended in 500 μL lysis buffer and sonicated with a Bioruptor sonication device (Diagenode) at 4°C with 30s on/off cycles at high intensity for 15 min. Three independent immunoprecipitations were performed for each sample as previously described and eluted DNA was purified using Qiagen PCR Purification columns. DNA quantification performed with custom primers and hydrolysis probes containing 5’ 6-FAM labels and ZEN (internal) and Iowa Black (3’) fluorescent quenchers (Integrated DNA Technologies) (table S4). qPCR was performed with two technical replicates for each sample and run on a LightCycler 480 (Roche) using TaqMan Universal PCR Master Mix (Thermo Fisher Scientific). Fold enrichment at four separate loci was determined using the delta-delta CT method by normalizing to a *dinG* control sequence (where CASP-σ does not bind) and to input DNA.

### De novo CASP-σ motif prediction

CASP-σ from the Csx30-CASP-σ structure predicted from Colabfold was structurally aligned in PyMol (Schrödinger) separately to the σ2 and σ4 domains of *E. coli* RpoE (PDB code: 1OR7) (49). Using the *E. coli* structure as a guide, sequence alignments to other ECF sigma factors were generated and used as an input for binding motifs prediction using predictECF (https://github.com/horiatodor/predictECF) (23) in R. Scripts for analysis and generating figures can be found in the Zenodo repository.

### CASP-σ motif scanning

Motifs for scanning the DiCASP loci (NZ_BEXT01000001:1,366,660–1,387,005), promoters from the *D. ishimotonii* genome, and the full *D. ishimotonii* genome (NZ_BEXT01000001) for putative CASP-σ binding sites were based on the position probability matrix created from the 13 peaks from ChIP-seq. Promoters were extracted by taking the 100 bps upstream of each annotated CDS in a Jupyter Notebook. Positions with R_seq_ ≤ 1 were masked and replaced with the average background nucleotide frequencies of each query sequence to avoid spurious sequence preferences in the motif due to potential undersampling of ChIP-seq hits (50, 51). Query sequences and motifs were analyzed using FIMO (https://meme-suite.org/meme/tools/fimo, version 5.4.1) (52). Scripts for analysis and generating tables as well as the query motifs in simple MEME format and the query sequences in .fasta format can be found in the Zenodo repository.

### Bacterial transcriptional reporters

Fluorescent transcriptional reporters were constructed by placing putative CASP-σ promoters upstream of msGFP in low copy pACYC plasmids. BL21 cells (Sigma Aldrich, CMC0016) were co-transformed with reporters and plasmids expressing CASP-σ, Csx30 isoforms, or empty controls and grown overnight in Terrific Broth. Cultures were diluted 1:10 in fresh media and GFP fluorescence measured in a Synergy Neo2 plate reader (BioTek, 488/528nm filter). The optical density at 600 nm was also read for each well and GFP levels normalized to cell density. Experiments were performed with 3 independent cultures for each condition.

### Structural predictions and homology searches

Csx30 and Csx30-CASP-σ structures were predicted using Colabfold (53), an interface for Alphafold2 (40) and MMSeqs2 (UniRef + environmental). Protein homology was determined using HHpred (54).

### Cell culture and transfection

HEK293T and Neuro2A cells were cultured in Dulbecco’s modified Eagle medium with high glucose, sodium pyruvate, and GlutaMAX (Thermo Fisher Scientific), 1× penicillin–streptomycin (Thermo Fisher Scientific), and 10% fetal bovine serum (Seradigm). Cells were maintained at a confluency below 90%. For immunoblot analysis, 24-well plates were seeded with 87,500 cells/well approximately 16 h before transfection. Cell were typically transfected with 50 ng of 3xHA-Csx30, 400 ng gRAMP, 400 ng CHAT, 100 ng target, and 500 ng crRNA in Opti-MEM (Thermo Fisher Scientific) with 4.5 μL TransIt-LT1 transfection reagent (Mirus). Spacer sequences for transcripts are listed in table S5.

For flow cytometry experiments, 96-well plates were seeded with 17,500 cells/well. Cell were typically transfected with 60 ng gRAMP, 60 ng CHAT, 20 ng target, 60 ng crRNA, and 0.5–5 ng of Cre constructs in Opti-MEM (Thermo Fisher Scientific) with 0.6 μL TransIt-LT1 transfection reagent (Mirus).

### Western blot and flow cytometry

Cells were typically harvested 96 h post-transfection. Cells were washed with ice-cold PBS and lysed in 75 μL of NP-40 lysis buffer (50 mM Tris pH 8, 150 mM NaCl, 1% NP-40). Cell suspensions were kept on ice for 10 min and cleared by centrifugation at 4C for 10 min at 21,000g. Lysates were stored at −80 before western blot analysis. Lysates were mixed with 4x Lammli buffer (Bio-Rad) run on 12 -well Nupage 4–12% Bis-Tris gels (Invitrogen). Proteins were transferred to PDVF membranes using an iBlot2 at 23V for 6 min. Membranes were blocked for 30 min at room temperature with TBST (Tris-buffer saline with 0.1% Tween 20) with 5% bovine serum albumin (Rockland). anti-HA:HRP (Cell Signaling Technologies, #2999) and anti-GAPDH:HRP (Cell Signaling Technologies #3683) were added at 1:5000 dilution and incubated for 30–60 min at room temperature. Membranes were washed 5x with TBST, incubated with Pierce ECL Western Blotting Substrate (Thermo Fisher Scientific) and imaged using a Chemi-Doc (Bio-Rad).

Immunoblots of *E. coli* cell lysates were performed in a similar manner. Cell input was normalized using optical density at 600 nm, and cell pellets were resuspended and lysed directly in Laemmli buffer.

Csx30 cleavage efficiency in immunoblots was estimated using image analysis in FIJI (55). The average signal intensity of each band was determined using a constant area selection and the lane background subtracted. Csx30 cleavage for each guide was determined as Csx30_cleaved_/(Csx30_cleaved_ +Csx30_full-length_ in three independent experiments. Expression levels of endogenous transcripts were determined from available HEK293T RNA-seq data (NCBI GEO database (56), accession GSE204833).

For flow cytometry analysis, cells were trypsinized 96 h post-transfection and resuspended in PBS supplemented with 5% FBS. Cells were analyzed using a CytoFLEX S flow cytometer (Beckman Coulter).

## Supplementary Material

Tables S1 - S7

Figures S1 - S28

## Data Availability

ChIP-seq data is available at the NCBI Sequence Read Archive (BioProject ID: PRJNA888197, SAMN31210314–21). Code and processed data is available at Zenodo (35). Plasmids are available from Addgene. The cryo-EM maps have been deposited in the Electron Microscopy Data Bank with the following codes: EMD-28064 (inactive CASP, focused refinement of Cas7–11, crRNA, and Csx29 NTD), EMD-28070 (inactive CASP, focused refinement of Csx29 TPR-CHAT), EMD-28065 (active CASP, focused refinement of Cas7–11, except INS, crRNA, and target RNA), EMD-28071 (active CASP, focused refinement of Cas7–11 INS), EMD-28072 (active CASP, focused refinement of Csx29 NTD and TPR), and EMD-28073 (active CASP, focused refinement of Csx29 CHAT and Csx30). The coordinates for the composite atomic models have been deposited in the Protein Data Bank under accession codes 8EEX (inactive CASP) and 8EEY (active CASP).

## References

[1] Bernheim A, Sorek R, The pan-immune system of bacteria: antiviral defence as a community resource. Nat. Rev. Microbiol. 18, 113–119 (2020). 31695182 10.1038/s41579-019-0278-2

[2] Gao L, Altae-Tran H, Böhning F, Makarova KS, Segel M, Schmid-Burgk JL, Koob J, Wolf YI, Koonin EV, Zhang F, Diverse enzymatic activities mediate antiviral immunity in prokaryotes. Science. 369, 1077–1084 (2020). 32855333 10.1126/science.aba0372 PMC7985843

[3] Makarova KS, Wolf YI, Iranzo J, Shmakov SA, Alkhnbashi OS, Brouns SJJ, Charpentier E, Cheng D, Haft DH, Horvath P, Moineau S, Mojica FJM, Scott D, Shah SA, Siksnys V, Terns MP, Venclovas Č, White MF, Yakunin AF, Yan W, Zhang F, Garrett RA, Backofen R, van der Oost J, Barrangou R, Koonin EV, Evolutionary classification of CRISPR-Cas systems: a burst of class 2 and derived variants. Nat. Rev. Microbiol. 18, 67–83 (2020). 31857715 10.1038/s41579-019-0299-x PMC8905525

[4] Shmakov SA, Makarova KS, Wolf YI, Severinov KV, Koonin EV, Systematic prediction of genes functionally linked to CRISPR-Cas systems by gene neighborhood analysis. Proc. Natl. Acad. Sci. U. S. A. 115, E5307–E5316 (2018). 29784811 10.1073/pnas.1803440115 PMC6003329

[5] Shah SA, Alkhnbashi OS, Behler J, Han W, She Q, Hess WR, Garrett RA, Backofen R, Comprehensive search for accessory proteins encoded with archaeal and bacterial type III CRISPR-cas gene cassettes reveals 39 new cas gene families. RNA Biol. 16, 530–542 (2019). 29911924 10.1080/15476286.2018.1483685 PMC6546367

[6] Peters JE, Makarova KS, Shmakov S, Koonin EV, Recruitment of CRISPR-Cas systems by Tn7-like transposons. Proc. Natl. Acad. Sci. U. S. A. 114, E7358–E7366 (2017). 28811374 10.1073/pnas.1709035114 PMC5584455

[7] Faure G, Shmakov SA, Yan WX, Cheng DR, Scott DA, Peters JE, Makarova KS, Koonin EV, CRISPR-Cas in mobile genetic elements: counter-defence and beyond. Nat. Rev. Microbiol. 17, 513–525 (2019). 31165781 10.1038/s41579-019-0204-7 PMC11165670

[8] Strecker J, Ladha A, Gardner Z, Schmid-Burgk JL, Makarova KS, Koonin EV, Zhang F, RNA-guided DNA insertion with CRISPR-associated transposases. Science. 365, 48–53 (2019). 31171706 10.1126/science.aax9181 PMC6659118

[9] Klompe SE, Vo PLH, Halpin-Healy TS, Sternberg SH, Transposon-encoded CRISPR-Cas systems direct RNA-guided DNA integration. Nature. 571, 219–225 (2019). 31189177 10.1038/s41586-019-1323-z

[10] Koonin EV, Makarova KS, Evolutionary plasticity and functional versatility of CRISPR systems. PLoS Biol. 20, e3001481 (2022). 34986140 10.1371/journal.pbio.3001481 PMC8730458

[11] Rouillon C, Schneberger N, Chi H, Peter MF, Geyer M, Boenigk W, Seifert R, White MF, Hagelueken G, SAVED by a toxin: Structure and function of the CRISPR Lon protease. bioRxiv. (2021), p. 2021.12.06.471393.

[12] Özcan A, Krajeski R, Ioannidi E, Lee B, Gardner A, Makarova KS, Koonin EV, Abudayyeh OO, Gootenberg JS, Programmable RNA targeting with the single-protein CRISPR effector Cas7–11. Nature. 597, 720–725 (2021). 34489594 10.1038/s41586-021-03886-5

[13] van Beljouw SPB, Haagsma AC, Rodríguez-Molina A, van den Berg DF, Vink JNA, Brouns SJJ, The gRAMP CRISPR-Cas effector is an RNA endonuclease complexed with a caspase-like peptidase. Science. 373, 1349–1353 (2021). 34446442 10.1126/science.abk2718

[14] van der Oost J, van der Oost J, Westra ER, Jackson RN, Wiedenheft B, Unravelling the structural and mechanistic basis of CRISPR–Cas systems. Nature Reviews Microbiology. 12 (2014), pp. 479–492. 24909109 10.1038/nrmicro3279 PMC4225775

[15] Aravind L, Koonin EV, Classification of the caspase-hemoglobinase fold: detection of new families and implications for the origin of the eukaryotic separins. Proteins. 46, 355–367 (2002). 11835511 10.1002/prot.10060

[16] Kato K, Zhou W, Okazaki S, Isayama Y, Nishizawa T, Gootenberg JS, Abudayyeh OO, Nishimasu H, Structure and engineering of the type III-E CRISPR-Cas7–11 effector complex. Cell (2022), doi:10.1016/j.cell.2022.05.003. 35643083

[17] Boland A, Martin TG, Zhang Z, Yang J, Bai X-C, Chang L, Scheres SHW, Barford D, Cryo-EM structure of a metazoan separase–securin complex at near-atomic resolution. Nature Structural & Molecular Biology. 24 (2017), pp. 414–418. 10.1038/nsmb.3386 PMC5385133 28263324

[18] Lin Z, Luo X, Yu H, Structural basis of cohesin cleavage by separase. Nature. 532, 131–134 (2016). 27027290 10.1038/nature17402 PMC4847710

[19] You L, Ma J, Wang J, Artamonova D, Wang M, Liu L, Xiang H, Severinov K, Zhang X, Wang Y, Structure Studies of the CRISPR-Csm Complex Reveal Mechanism of Co-transcriptional Interference. Cell. 176, 239–253.e16 (2019). 30503210 10.1016/j.cell.2018.10.052 PMC6935017

[20] Sofos N, Feng M, Stella S, Pape T, Fuglsang A, Lin J, Huang Q, Li Y, She Q, Montoya G, Structures of the Cmr-β Complex Reveal the Regulation of the Immunity Mechanism of Type III-B CRISPR-Cas. Mol. Cell. 79, 741–757.e7 (2020). 32730741 10.1016/j.molcel.2020.07.008

[21] McLuskey K, Mottram JC, Comparative structural analysis of the caspase family with other clan CD cysteine peptidases. Biochem. J. 466, 219–232 (2015). 25697094 10.1042/BJ20141324 PMC4357240

[22] Feklístov A, Sharon BD, Darst SA, Gross CA, Bacterial sigma factors: a historical, structural, and genomic perspective. Annu. Rev. Microbiol. 68, 357–376 (2014). 25002089 10.1146/annurev-micro-092412-155737

[23] Todor H, Osadnik H, Campbell EA, Myers KS, Li H, Donohue TJ, Gross CA, Rewiring the specificity of extracytoplasmic function sigma factors. Proc. Natl. Acad. Sci. U. S. A. 117, 33496–33506 (2020). 33318184 10.1073/pnas.2020204117 PMC7776599

[24] OMP Peptide Signals Initiate the Envelope-Stress Response by Activating DegS Protease via Relief of Inhibition Mediated by Its PDZ Domain. Cell. 113, 61–71 (2003). 12679035 10.1016/s0092-8674(03)00203-4

[25] Schöbel S, Zellmeier S, Schumann W, Wiegert T, The Bacillus subtilis sigmaW anti-sigma factor RsiW is degraded by intramembrane proteolysis through YluC. Mol. Microbiol. 52, 1091–1105 (2004). 15130127 10.1111/j.1365-2958.2004.04031.x

[26] Ades SE, Connolly LE, Alba BM, Gross CA, The Escherichia coli sigma(E)-dependent extracytoplasmic stress response is controlled by the regulated proteolysis of an anti-sigma factor. Genes Dev. 13, 2449–2461 (1999). 10500101 10.1101/gad.13.18.2449 PMC317020

[27] Lane WJ, Darst SA, The Structural Basis for Promoter −35 Element Recognition by the Group IV σ Factors. PLoS Biology. 4 (2006), p. e269. 16903784 10.1371/journal.pbio.0040269 PMC1540707

[28] Casas-Pastor D, Müller RR, Jaenicke S, Brinkrolf K, Becker A, Buttner MJ, Gross CA, Mascher T, Goesmann A, Fritz G, Expansion and re-classification of the extracytoplasmic function (ECF) σ factor family. Nucleic Acids Res. 49, 986–1005 (2021). 33398323 10.1093/nar/gkaa1229 PMC7826278

[29] Gootenberg JS, Abudayyeh OO, Lee JW, Essletzbichler P, Dy AJ, Joung J, Verdine V, Donghia N, Daringer NM, Freije CA, Myhrvold C, Bhattacharyya RP, Livny J, Regev A, Koonin EV, Hung DT, Sabeti PC, Collins JJ, Zhang F, Nucleic acid detection with CRISPR-Cas13a/C2c2. Science. 356, 438–442 (2017). 28408723 10.1126/science.aam9321 PMC5526198

[30] Alba BM, Leeds JA, Onufryk C, Lu CZ, Gross CA, DegS and YaeL participate sequentially in the cleavage of RseA to activate the ς^E^-dependent extracytoplasmic stress response. Genes & Development. 16 (2002), pp. 2156–2168. 12183369 10.1101/gad.1008902 PMC186436

[31] Kanehara K, Ito K, Akiyama Y, YaeL (EcfE) activates the ςE pathway of stress response through a site-2 cleavage of anti-ςE, RseA. Genes & Development. 16 (2002), pp. 2147–2155. 12183368 10.1101/gad.1002302 PMC186437

[32] Flynn JM, Levchenko I, Sauer RT, Baker TA, Modulating substrate choice: the SspB adaptor delivers a regulator of the extracytoplasmic-stress response to the AAA+ protease ClpXP for degradation. Genes Dev. 18, 2292–2301 (2004). 15371343 10.1101/gad.1240104 PMC517522

[33] Ahator SD, Jianhe W, Zhang L-H, The ECF sigma factor PvdS regulates the type I-F CRISPR-Cas system in Pseudomonas aeruginosa. bioRxiv (2020), p. 2020.01.31.929752.

[34] Malone LM, Hampton HG, Morgan XC, Fineran PC, Type I CRISPR-Cas provides robust immunity but incomplete attenuation of phage-induced cellular stress. Nucleic Acids Res. 50, 160–174 (2022). 34928385 10.1093/nar/gkab1210 PMC8754663

[35] Strecker J, Li D, Zhang F Code and processed data for: RNA-activated protein cleavage with a CRISPR-associated endopeptidase (Version 1.0). Zenodo 10.5281/zenodo.7221526. PMC10028731 36423276

[36] Kimanius D, Dong L, Sharov G, Nakane T, Scheres SHW, New tools for automated cryo-EM single-particle analysis in RELION-4.0. Biochem J. 478, 4169–4185 (2021). 34783343 10.1042/BCJ20210708 PMC8786306

[37] Morin A, Eisenbraun B, Key J, Sanschagrin PC, Timony MA, Ottaviano M, Sliz P, Collaboration gets the most out of software. Elife. 2, e01456 (2013). 24040512 10.7554/eLife.01456 PMC3771563

[38] Rohou A, Grigorieff N, CTFFIND4: Fast and accurate defocus estimation from electron micrographs. J. Struct. Biol. 192, 216–221 (2015). 26278980 10.1016/j.jsb.2015.08.008 PMC6760662

[39] Bepler T, Morin A, Rapp M, Brasch J, Shapiro L, Noble AJ, Berger B, Positive-unlabeled convolutional neural networks for particle picking in cryo-electron micrographs. Nat. Methods. 16, 1153–1160 (2019). 31591578 10.1038/s41592-019-0575-8 PMC6858545

[40] Jumper J, Evans R, Pritzel A, Green T, Figurnov M, Ronneberger O, Tunyasuvunakool K, Bates R, Žídek A, Potapenko A, Bridgland A, Meyer C, Kohl SAA, Ballard AJ, Cowie A, Romera-Paredes B, Nikolov S, Jain R, Adler J, Back T, Petersen S, Reiman D, Clancy E, Zielinski M, Steinegger M, Pacholska M, Berghammer T, Bodenstein S, Silver D, Vinyals O, Senior AW, Kavukcuoglu K, Kohli P, Hassabis D, Highly accurate protein structure prediction with AlphaFold. Nature. 596, 583–589 (2021). 34265844 10.1038/s41586-021-03819-2 PMC8371605

[41] Casañal A, Lohkamp B, Emsley P, Current developments in Coot for macromolecular model building of Electron Cryo-microscopy and Crystallographic Data. Protein Sci. 29, 1069–1078 (2020). 31730249 10.1002/pro.3791 PMC7096722

[42] Croll TI, ISOLDE: a physically realistic environment for model building into low-resolution electron-density maps. Acta Crystallogr D Struct Biol. 74, 519–530 (2018). 29872003 10.1107/S2059798318002425 PMC6096486

[43] Liebschner D, Afonine PV, Baker ML, Bunkóczi G, Chen VB, Croll TI, Hintze B, Hung LW, Jain S, McCoy AJ, Moriarty NW, Oeffner RD, Poon BK, Prisant MG, Read RJ, Richardson JS, Richardson DC, Sammito MD, Sobolev OV, Stockwell DH, Terwilliger TC, Urzhumtsev AG, Videau LL, Williams CJ, Adams PD, Macromolecular structure determination using X-rays, neutrons and electrons: recent developments in Phenix. Acta Crystallogr D Struct Biol. 75, 861–877 (2019). 31588918 10.1107/S2059798319011471 PMC6778852

[44] Williams CJ, Headd JJ, Moriarty NW, Prisant MG, Videau LL, Deis LN, Verma V, Keedy DA, Hintze BJ, Chen VB, Jain S, Lewis SM, Arendall WB 3rd, Snoeyink J, Adams PD, Lovell SC, Richardson JS, Richardson DC, MolProbity: More and better reference data for improved all-atom structure validation. Protein Sci. 27, 293–315 (2018). 29067766 10.1002/pro.3330 PMC5734394

[45] Tan YZ, Baldwin PR, Davis JH, Williamson JR, Potter CS, Carragher B, Lyumkis D, Addressing preferred specimen orientation in single-particle cryo-EM through tilting. Nat. Methods. 14, 793–796 (2017). 28671674 10.1038/nmeth.4347 PMC5533649

[46] Schmid-Burgk JL, Hornung V, BrowserGenome.org: web-based RNA-seq data analysis and visualization. Nat. Methods. 12, 1001 (2015). 26513548 10.1038/nmeth.3615

[47] Bailey TL, Johnson J, Grant CE, Noble WS, The MEME Suite. Nucleic Acids Res. 43, W39–49 (2015). 25953851 10.1093/nar/gkv416 PMC4489269

[48] Tareen A, Kinney JB, Logomaker: beautiful sequence logos in Python. Bioinformatics. 36, 2272–2274 (2020). 31821414 10.1093/bioinformatics/btz921 PMC7141850

[49] Campbell EA, Tupy JL, Gruber TM, Wang S, Sharp MM, Gross CA, Darst SA, Crystal structure of Escherichia coli sigmaE with the cytoplasmic domain of its anti-sigma RseA. Mol. Cell. 11, 1067–1078 (2003). 12718891 10.1016/s1097-2765(03)00148-5

[50] Crooks GE, Hon G, Chandonia J-M, Brenner SE, WebLogo: a sequence logo generator. Genome Res. 14, 1188–1190 (2004). 15173120 10.1101/gr.849004 PMC419797

[51] Schneider TD, Stephens RM, Sequence logos: a new way to display consensus sequences. Nucleic Acids Res. 18, 6097–6100 (1990). 2172928 10.1093/nar/18.20.6097 PMC332411

[52] Grant CE, Bailey TL, Noble WS, FIMO: scanning for occurrences of a given motif. Bioinformatics. 27, 1017–1018 (2011). 21330290 10.1093/bioinformatics/btr064 PMC3065696

[53] Mirdita M, Schütze K, Moriwaki Y, Heo L, Ovchinnikov S, Steinegger M, ColabFold: making protein folding accessible to all. Nat. Methods. 19, 679–682 (2022). 35637307 10.1038/s41592-022-01488-1 PMC9184281

[54] Zimmermann L, Stephens A, Nam S-Z, Rau D, Kübler J, Lozajic M, Gabler F, Söding J, Lupas AN, Alva V, A Completely Reimplemented MPI Bioinformatics Toolkit with a New HHpred Server at its Core. J. Mol. Biol. 430, 2237–2243 (2018). 29258817 10.1016/j.jmb.2017.12.007

[55] Schindelin J, Arganda-Carreras I, Frise E, Kaynig V, Longair M, Pietzsch T, Preibisch S, Rueden C, Saalfeld S, Schmid B, Tinevez J-Y, White DJ, Hartenstein V, Eliceiri K, Tomancak P, Cardona A, Fiji: an open-source platform for biological-image analysis. Nature Methods. 9 (2012), pp. 676–682. 22743772 10.1038/nmeth.2019 PMC3855844

[56] Lim CKW, McCallister TX, Saporito-Magriña C, McPheron GD, Krishnan R, Zeballos C MA, Powell JE, Clark LV, Perez-Pinera P, Gaj T, CRISPR base editing of cis-regulatory elements enables the perturbation of neurodegeneration-linked genes. Mol. Ther. (2022), doi:10.1016/j.ymthe.2022.08.008. PMC9734028 35965414

