# Supplementary material for: RNA-activated protein cleavage with a CRISPR-associated endopeptidase
Source: Science. Author manuscript; Available in PMC 2023 Mar 21. (PMC10028731; doi:10.1126/science.add7450)
Supplement: Figures S1 - S28 [file NIHMS1863051-supplement-Figures_S1_-_S28.pdf]

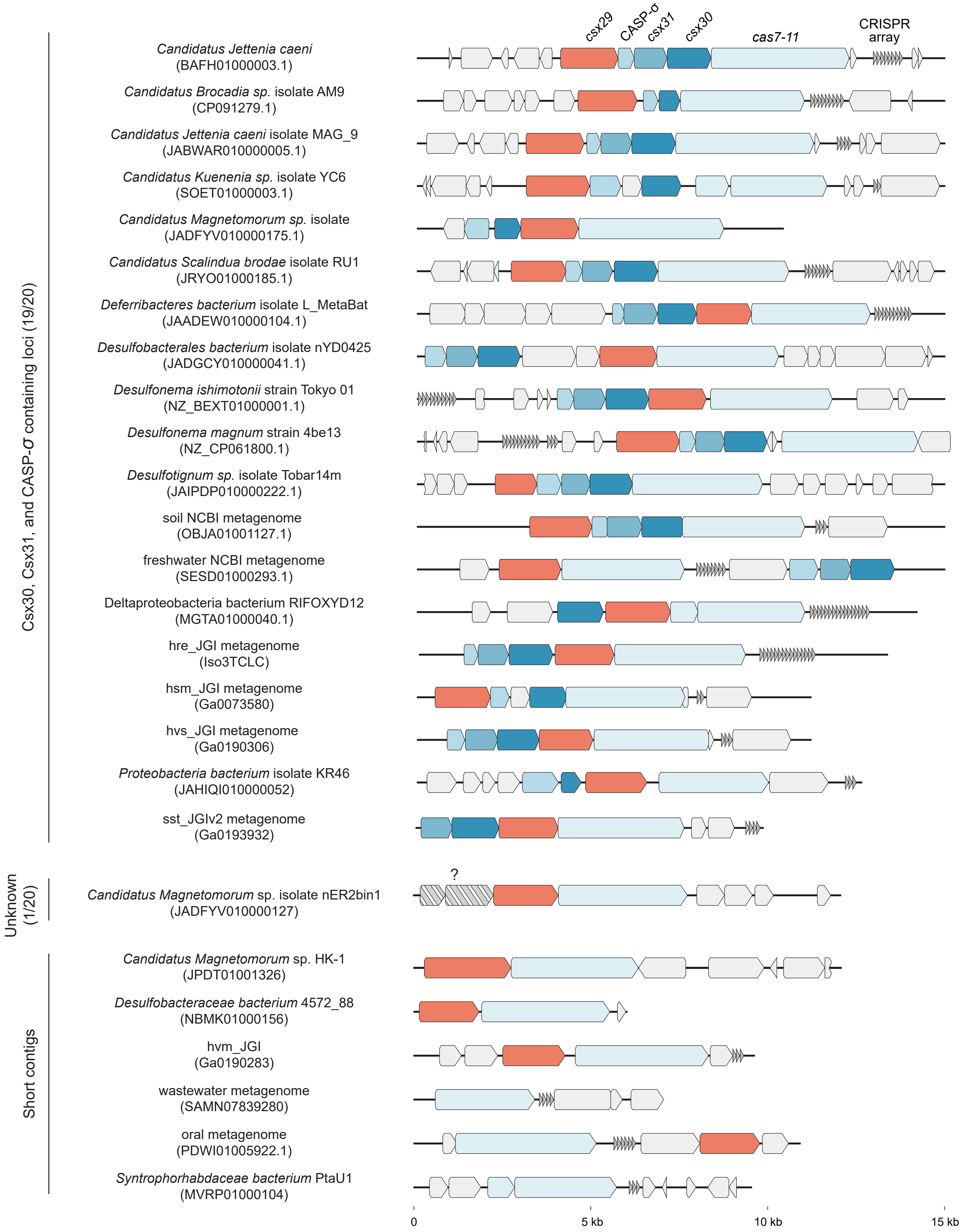

Fig. S1. Schematic of type III-E CRISPR loci in nature and the prevalence of associated *csx30*, *csx31*, and CASP-σ genes. 19 of 20 loci contain at least two of the three genes while several contigs are too short to confidently assess.

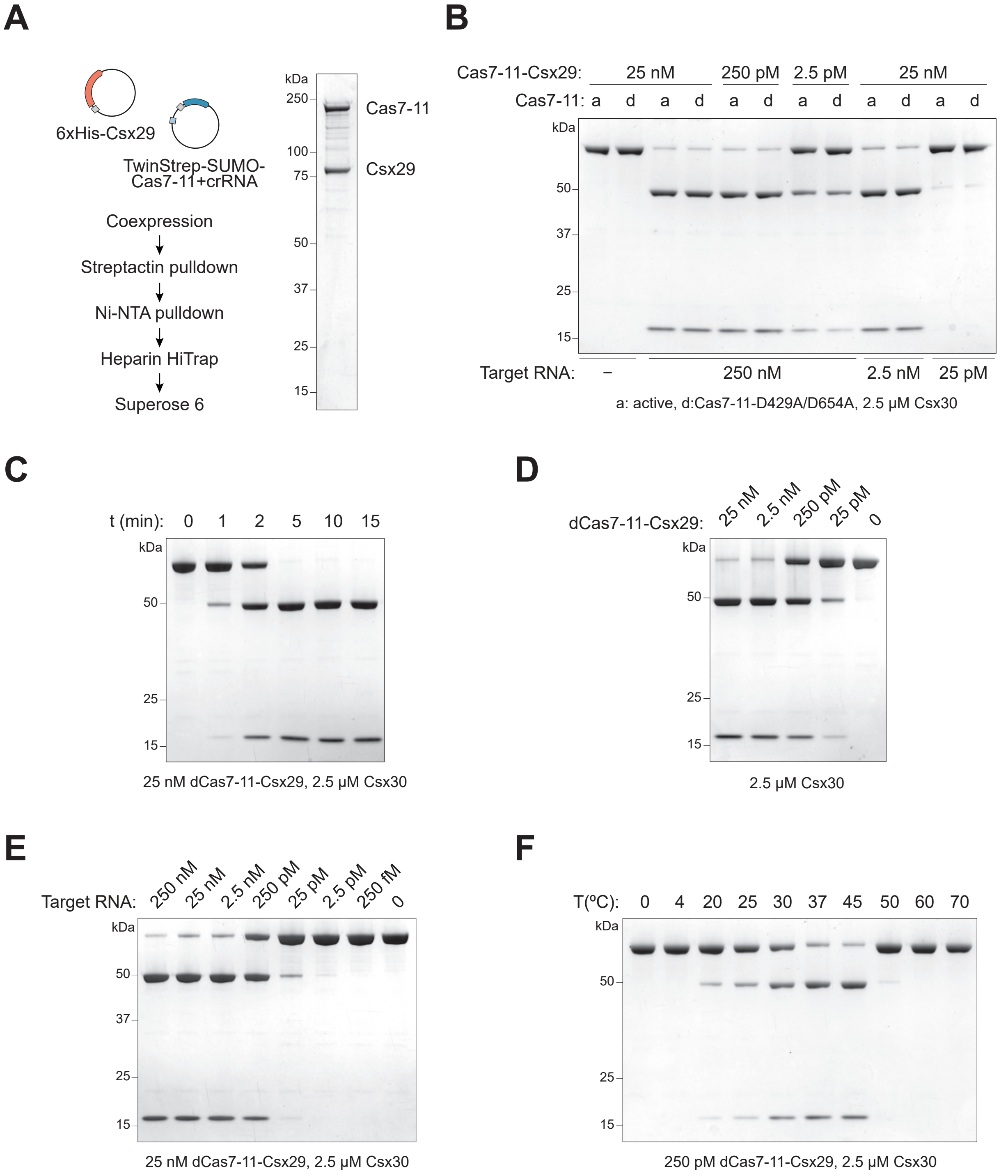

Fig. S2. In vitro characterization of Cas7-11-Csx29 proteolytic activity on Csx30. (**A**) Purification schematic and SDS-PAGE analysis of a Cas7-11-Csx29 complex. (**B**) Comparison of Csx30 cleavage by Csx29 and nuclease active and dead Cas7-11. (**C**) Time course of Csx30 cleavage upon addition of target RNA. (**D**) Dilution series of Cas7-11-Csx29 relative to Csx30 concentration. (**E**) Csx30 cleavage across dilution series of target RNA. (**F**) Csx30 cleavage across a temperature range. Panels A-E are SDS-PAGE gels stained with Coomassie. Panels C-F were performed with catalytically inactive dCas7-11.

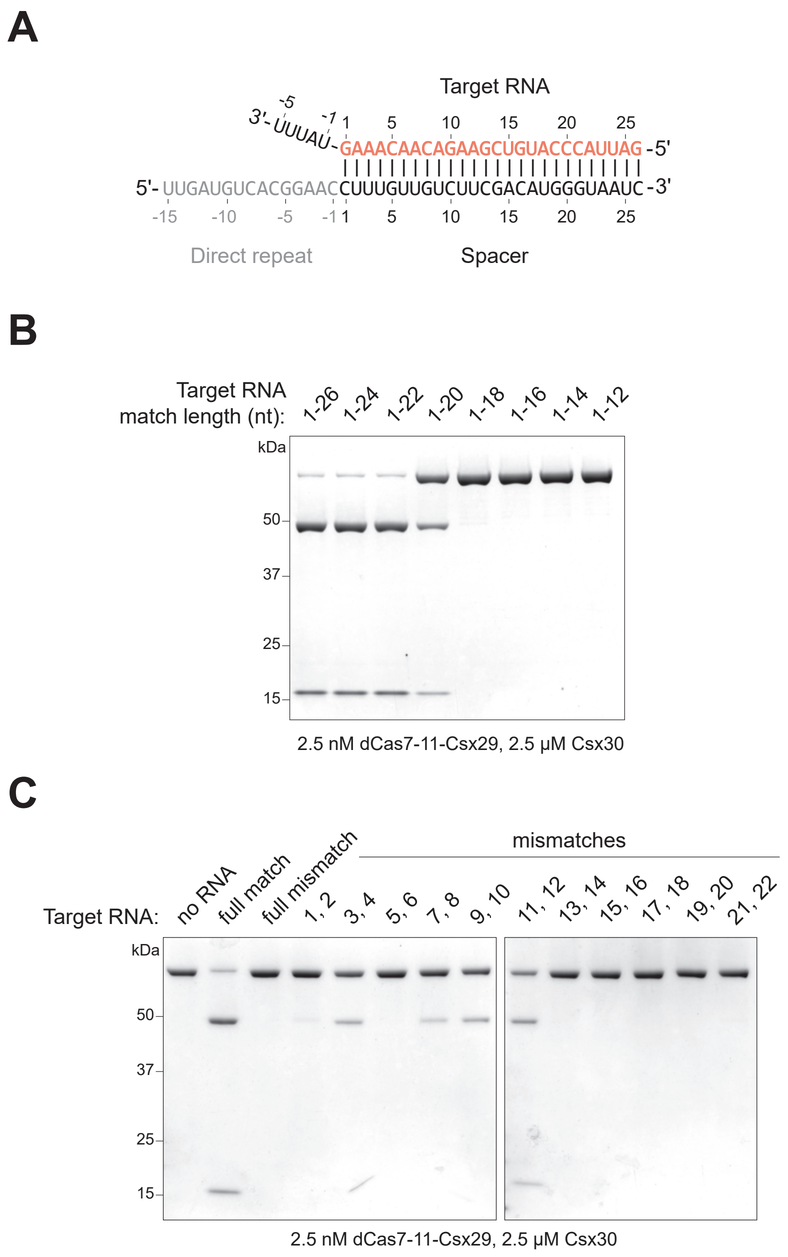

Fig. S3. In vitro characterization of target RNA requirements for Csx30 cleavage. (**A**) Schematic of the crRNA co-expressed with Cas7-11-Csx29 with the complementary region of the target RNA being modified highlighted in red. (**B**) Length requirement of crRNA-target RNA complementarity required for Csx30 cleavage. All target RNA were kept at the same physical length and mismatch substitutions were introduced to prevent target RNA-crRNA annealing. (**C**) Csx30 cleavage using target RNAs that contain base pair mismatches. Mutations were generated to match the corresponding position in the crRNA.

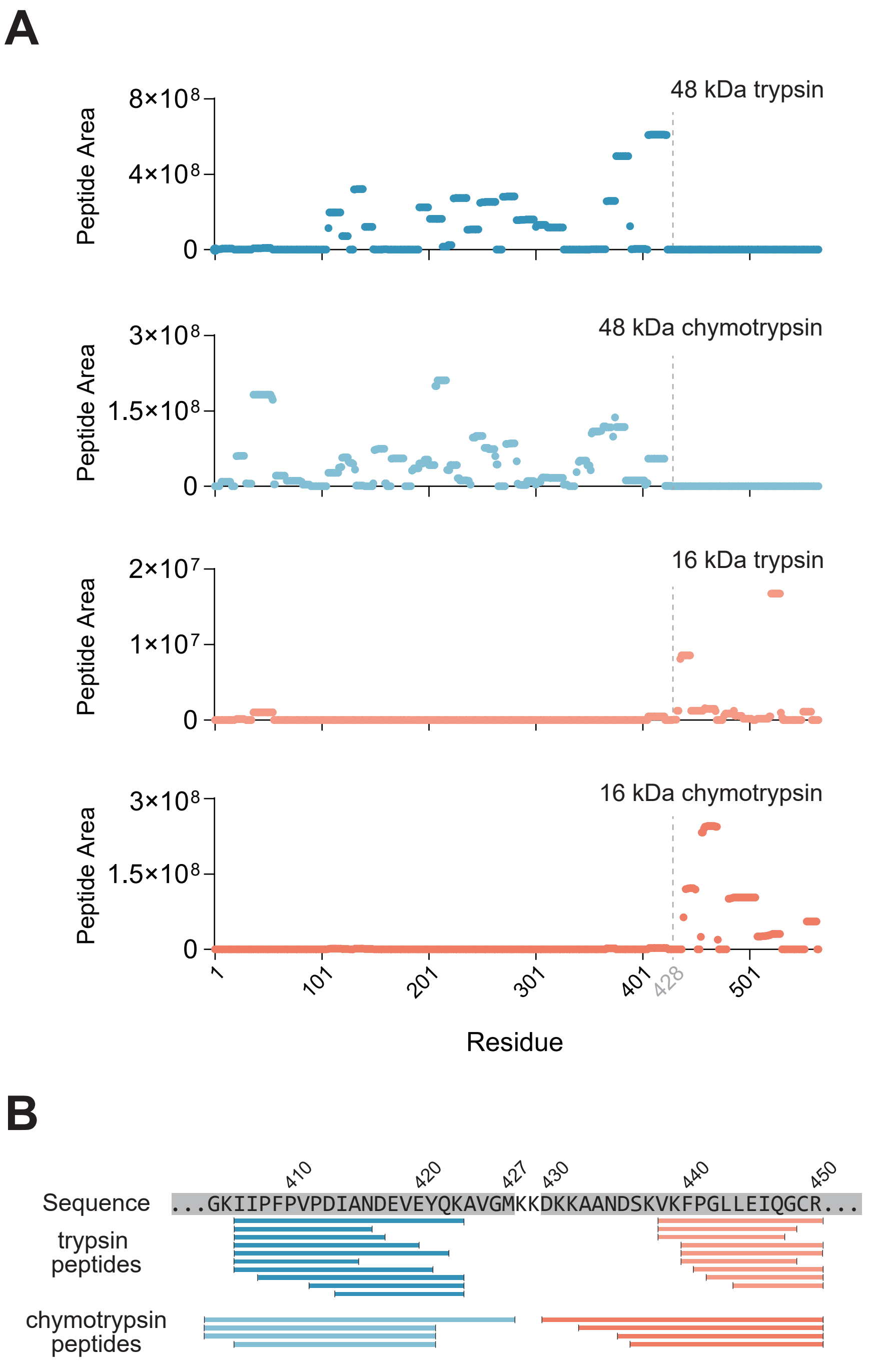

Fig. S4. Identification of the Csx30 cleavage site. (**A**) Mass spectrometry analysis of the Csx30 processed fragments following trypsin and chymotrypsin digests. (**B**) Unique peptides detected by mass spectrometry around the Csx30 cleavage site.

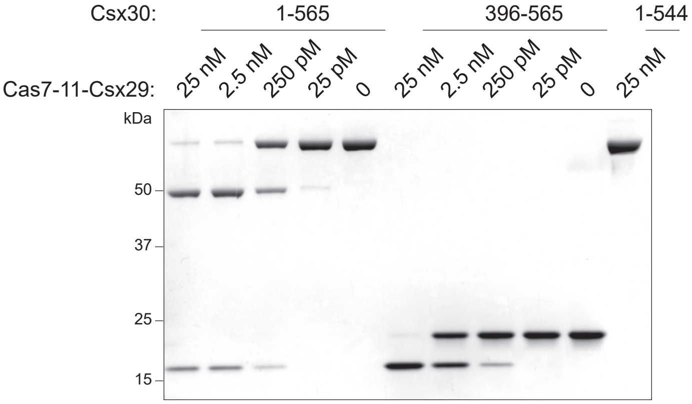

Fig. S5. In vitro cleavage of truncated Csx30 proteins. SDS-PAGE gel stained with Coomassie.

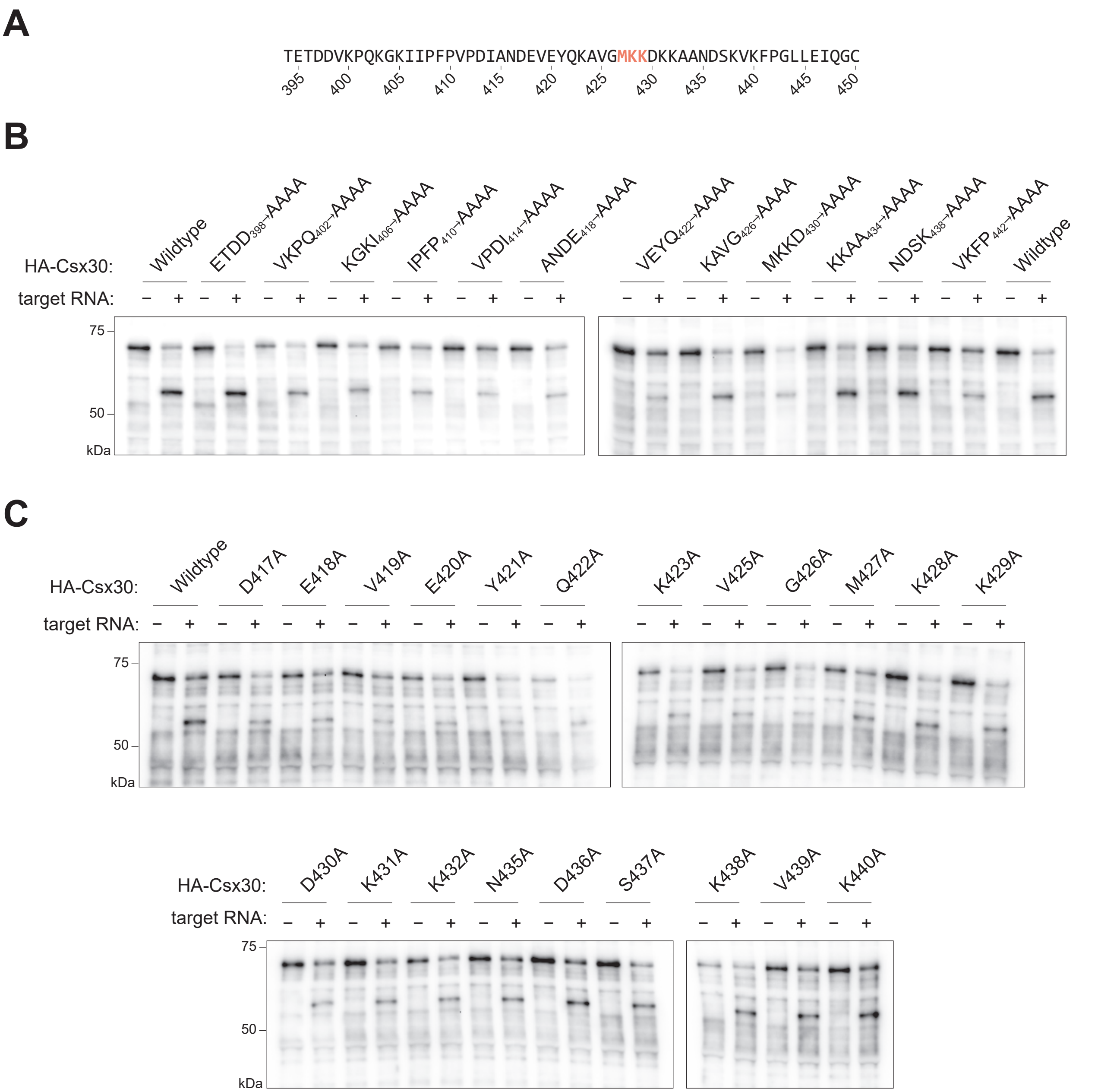

Fig. S6. Alanine scanning mutagenesis of Csx30. (**A**) Csx30 residues surrounding the cleavage site. (**B**) Immunoblot analysis of in vitro reactions with N-terminal HA-tagged Csx30 quadruple alanine mutants produced by cell-free transcription-translation. (**C**) Immunoblot analysis of in vitro reactions with N-terminal HA-tagged Csx30 single alanine mutants produced by cell-free transcription-translation.

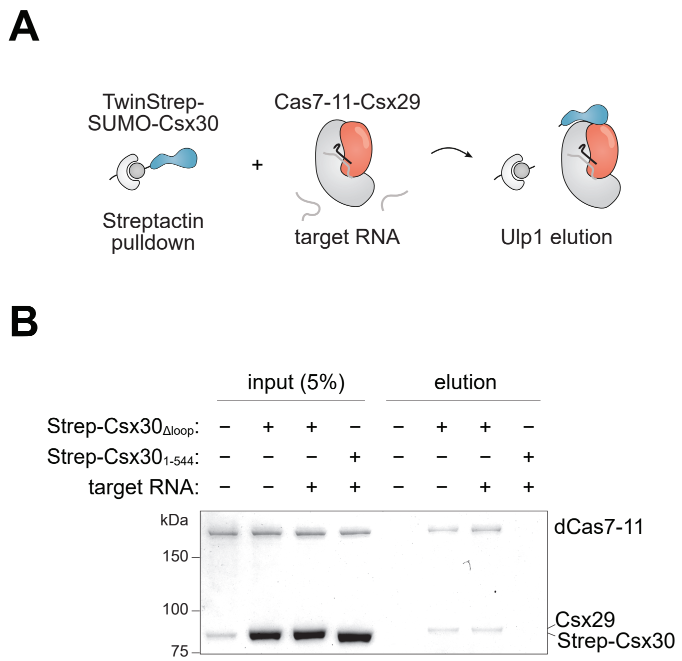

Fig. S7. Characterization of Csx30 binding to Cas7-11-Csx29. (**A**) Schematic of Csx30 pulldown experiments. (**B**) dCas7-11-Csx29 binds to $Csx30_{\Delta loop}$ independent of target RNA, but does not bind to $Csx30_{1\text{-}544}$. SDS-PAGE gels stained with Coomassie following elution with the SUMO protease Ulp1.

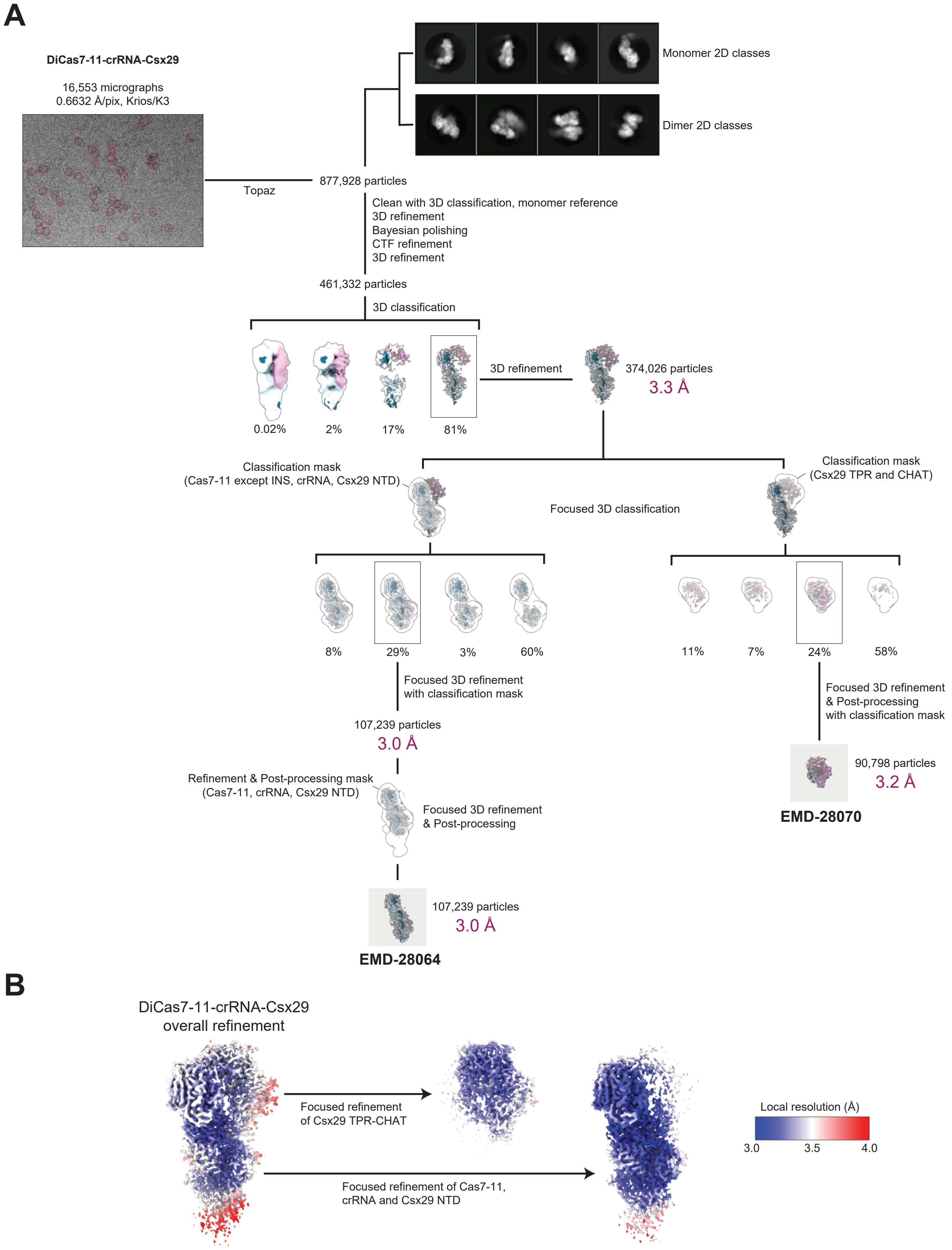

Fig. S8. Single particle reconstruction of DiCas7-11-crRNA-Csx29 complex. (**A**) Cryo-EM data processing workflow. Final maps deposited to the EMDB are highlighted. (**B**) Sharpened EM density maps colored by local resolution as calculated by RELION.

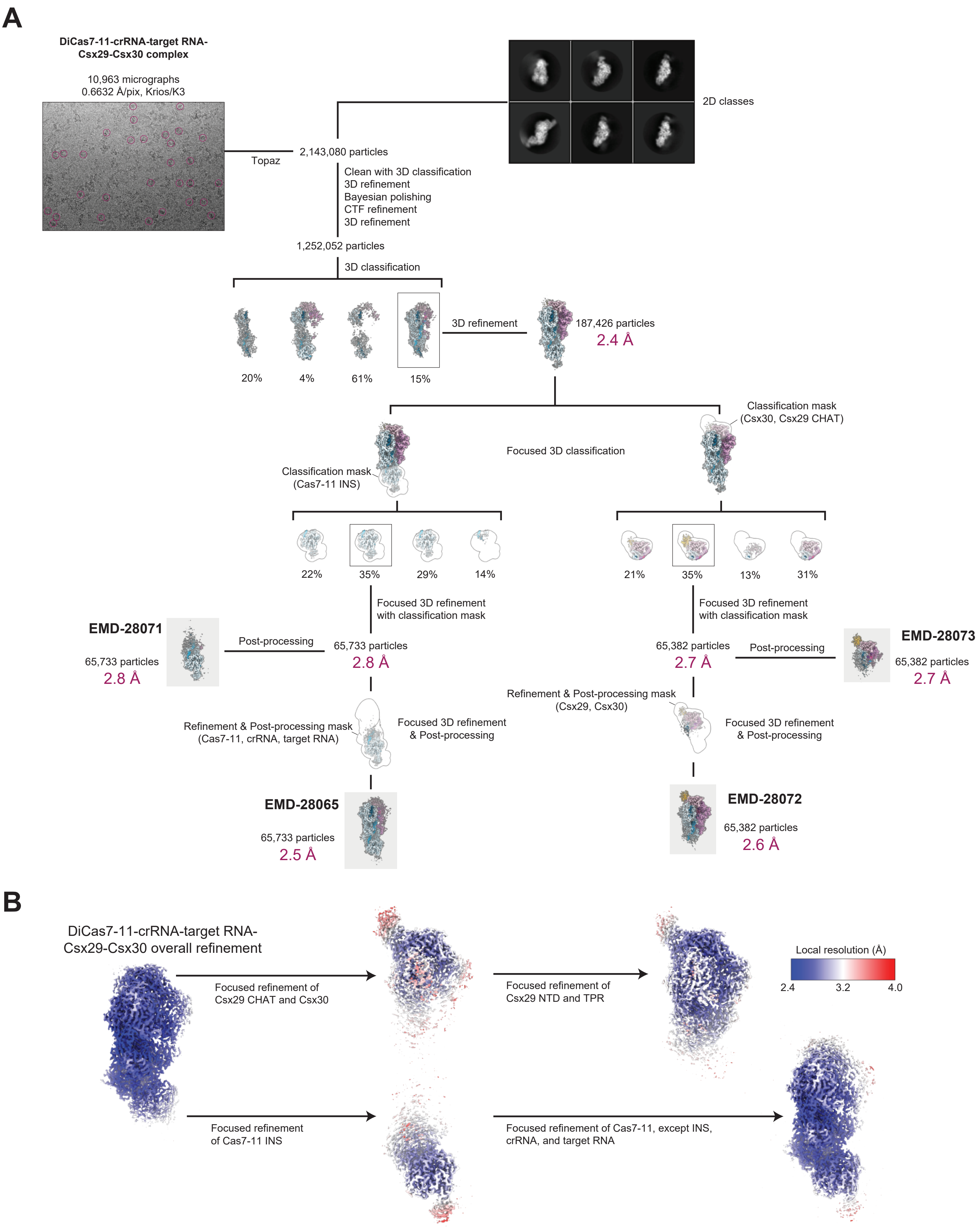

Fig. S9. Single particle reconstruction of DiCas7-11-crRNA-target RNA-Csx29-Csx30 complex. (**A**) Cryo-EM data processing workflow. Final maps deposited to the EMDB are highlighted. (**B**) Sharpened EM density maps colored by local resolution as calculated by RELION.

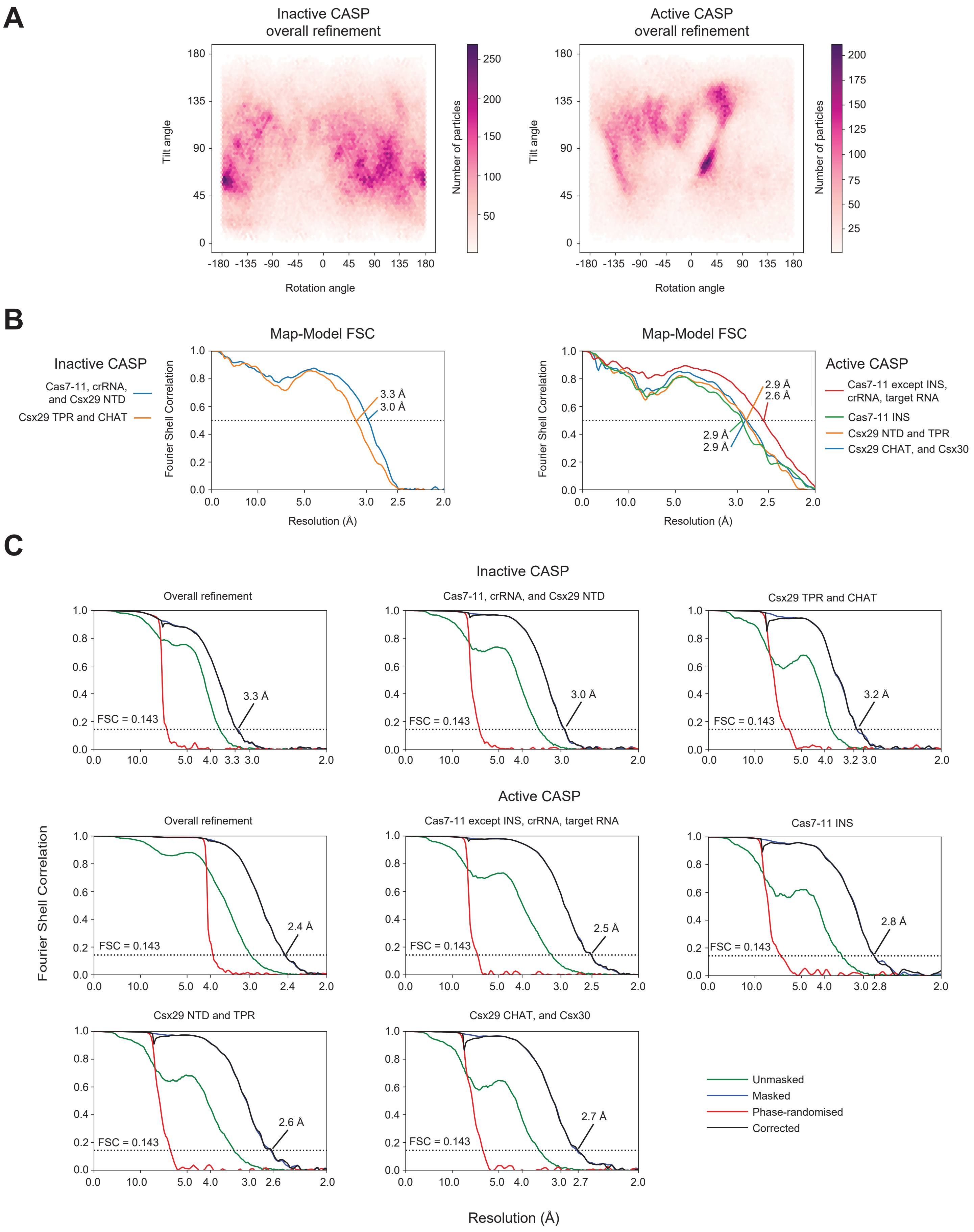

Fig. S10. Cryo-EM data statistics. (**A**) Orientation distribution for reconstructions of the CASP complex in inactive and active states. (**B**) Map-to-model Fourier-Shell Correlation for each model, calculated by softly masking each map around the fitted model. (**C**) Gold-standard Fourier-Shell Correlation curves.

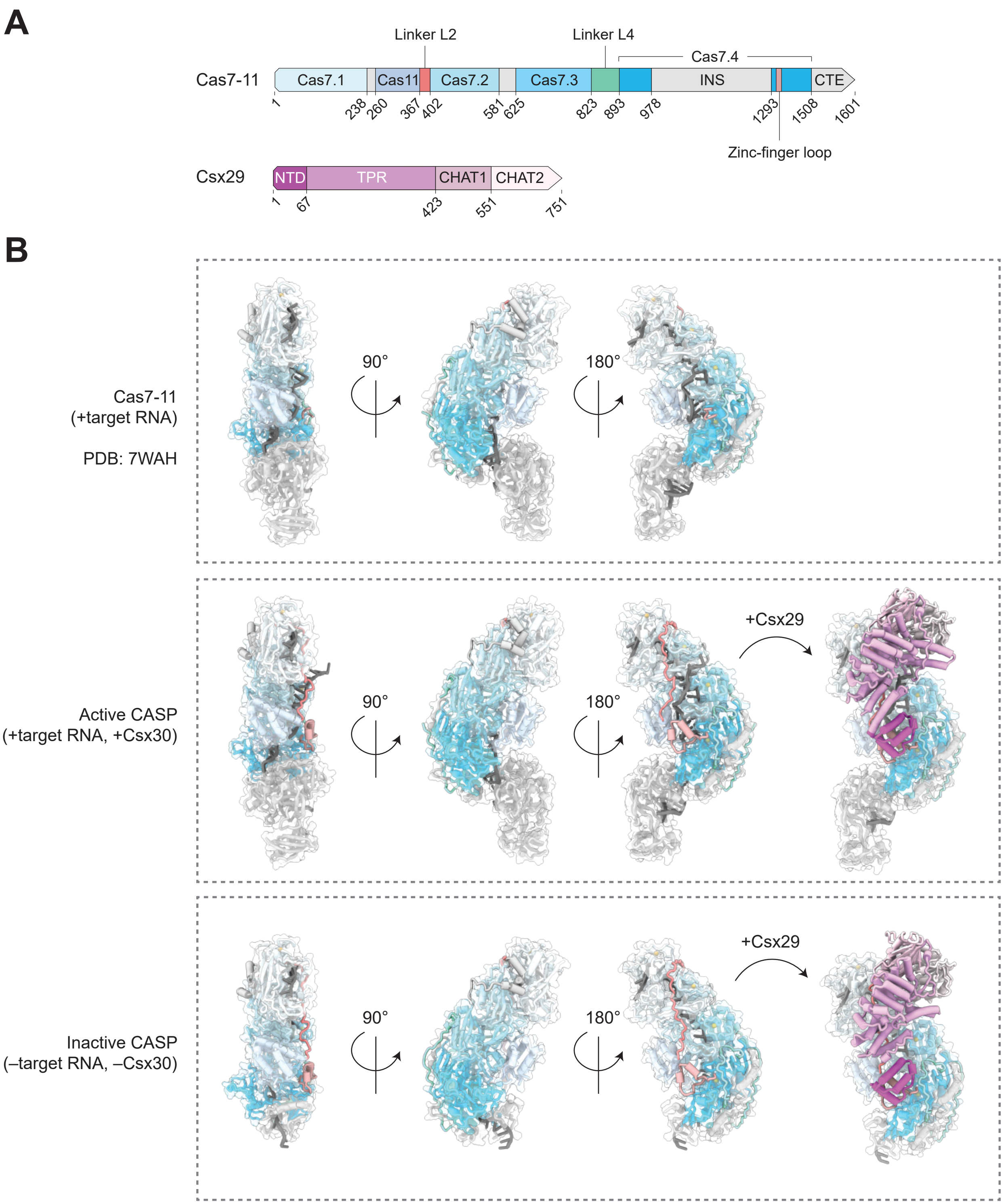

Fig. S11. Comparison of Cas7-11 overall architecture in different states. (**A**) Schematic of Cas7-11, and Csx29 protein domains (**B**) Overall views of Cas7-11 in apo- and CASP states with corresponding domain coloring as in panel A. crRNA and target RNA are both colored in dark gray. Upon Csx29 binding, Cas7-11 linker L2 becomes structured, and makes contacts with target RNA and Csx29. Also, a short region (aa 1313-1340) extending from the zinc-finger of Cas7.4 forms a coiled-coil, and stacks against Csx29 NTD. Cas7.2-Cas7.4 resides at the Csx29 interface contacting NTD, TPR and CHAT1 domains. Unlike linker L2, linker L4 does not structurally change upon Csx29 interaction.

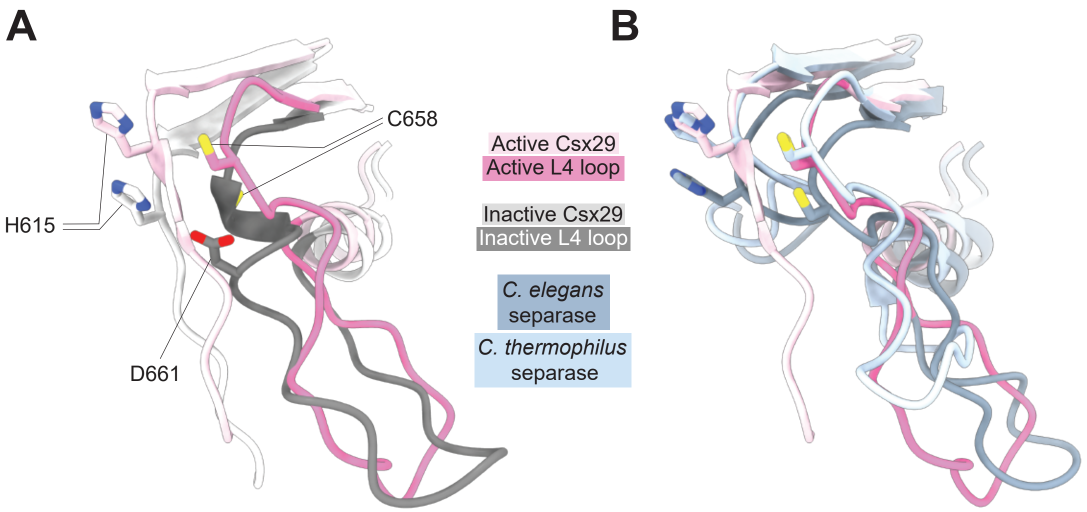

Fig. S12. Comparison of the Csx29 catalytic site with other caspases. (**A**) Superposed Csx29 structures in the inactive and active states. The L4 loop containing the catalytic cysteine is colored darker in both structures. (**B**) The active Csx29 structure superposed on *Caenorhabditis elegans* separase (PDB: 5MZ6) and *Chaetomium thermophilum* separase (PDB: 5FBY). The L4 loop of activated Csx29 adopts a similar shape to caspases, exposing C658 toward H615.

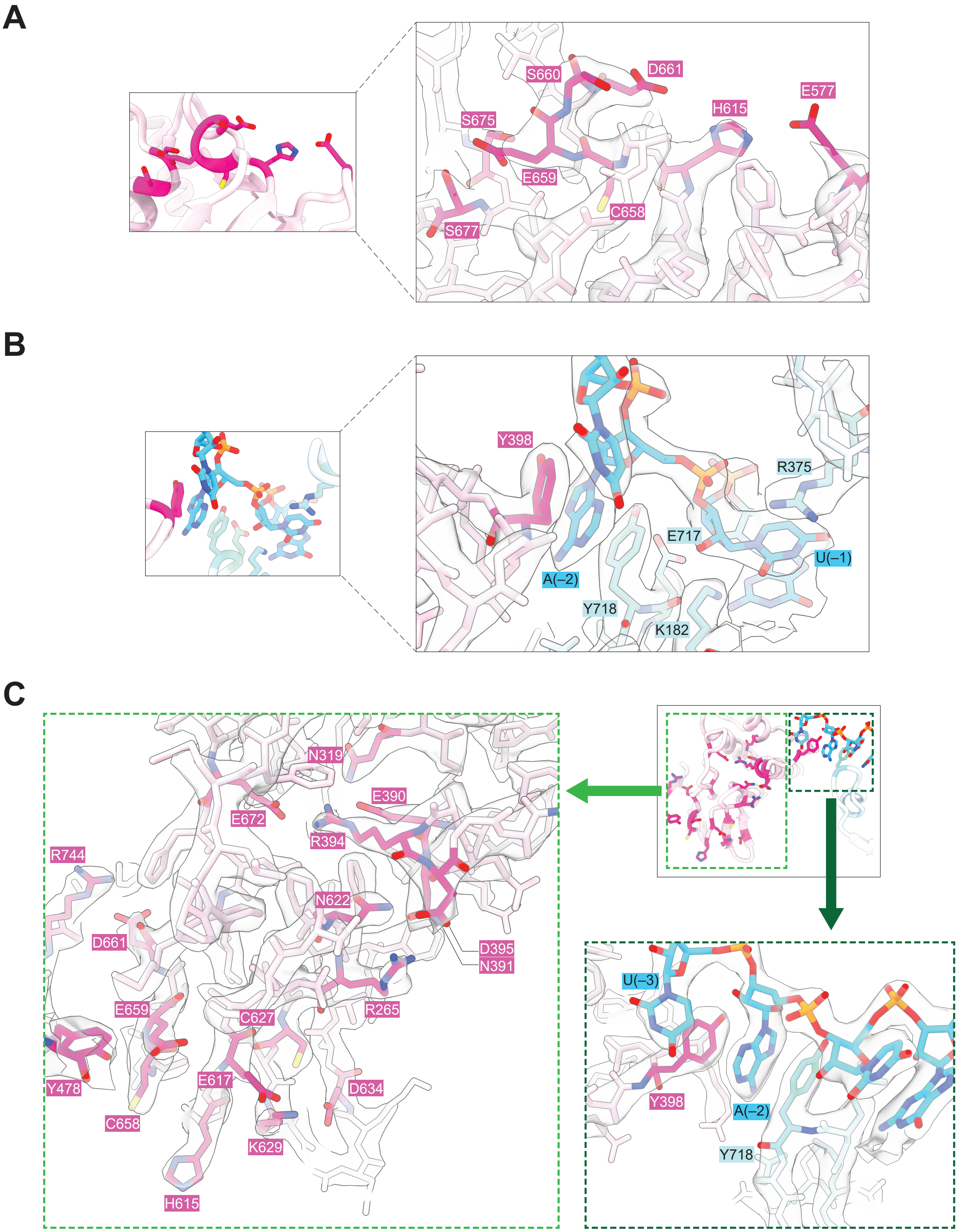

Fig. S13. Allosteric activation of CASP. (**A**) Electrostatic and hydrogen bonded network within the Csx29 catalytic site in the inactive state, as in Fig. 2D, shown with corresponding EM density. (**B**) Contacts between Cas7-11 and the DR-mismatched portion of the target RNA in the active state, as in Fig. 2G, shown with corresponding EM density. (**C**) Electrostatic and hydrogen bonded network extending from the AR to the Csx29 catalytic site in the active state, as in Fig. 2H, shown with corresponding EM density.

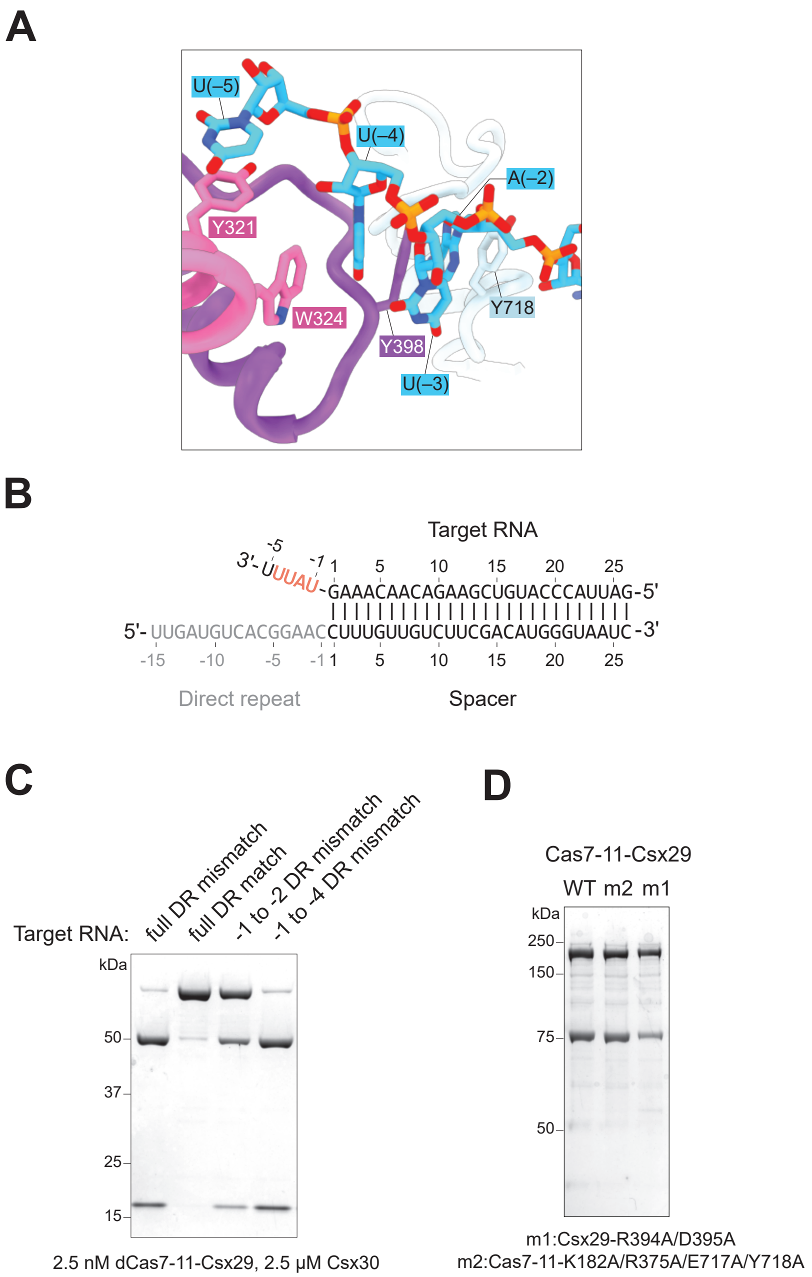

Fig. S14. Characterization of Cas7-11-Csx29 proteolytic activity using DR complementary target RNA. (**A**) Cas7-11, and Csx29 AR residues which mediate base stacking interactions with the target RNA are shown: Y398/U(–3)/Y718, U(–4)/W324, U(–5)/Y321. (**B**) Schematic of the crRNA co-expressed with Cas7-11-Csx29 and the 3’ region of the target RNA being modified highlighted in red. (**C**) Csx30 cleavage using target RNA with different degrees of DR complementarity. (**D**) SDS-PAGE gel stained with Coomassie of activation mutant Cas7-11-Csx29 complexes.

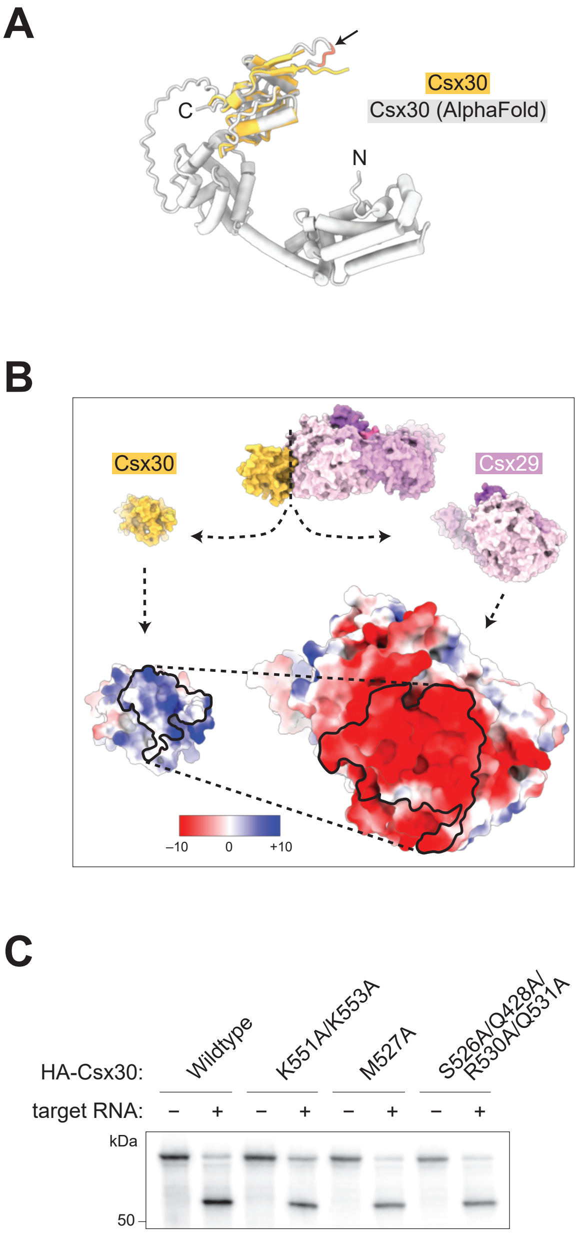

Fig. S15. Structural analysis of Csx30 recognition by Csx29. (**A**) Structurally characterized portion of Csx30 is superposed on the AlphaFold2 model. The predicted cleavage site is colored red and indicated with an arrow. (**B**) Electrostatic surface potential of the Csx29-Csx30 interface within the active CASP complex. (**C**) Immuno-blot analysis of in vitro cleavage reactions with N-terminal HA-tagged Csx30 alanine mutants produced by cell-free transcription-translation.

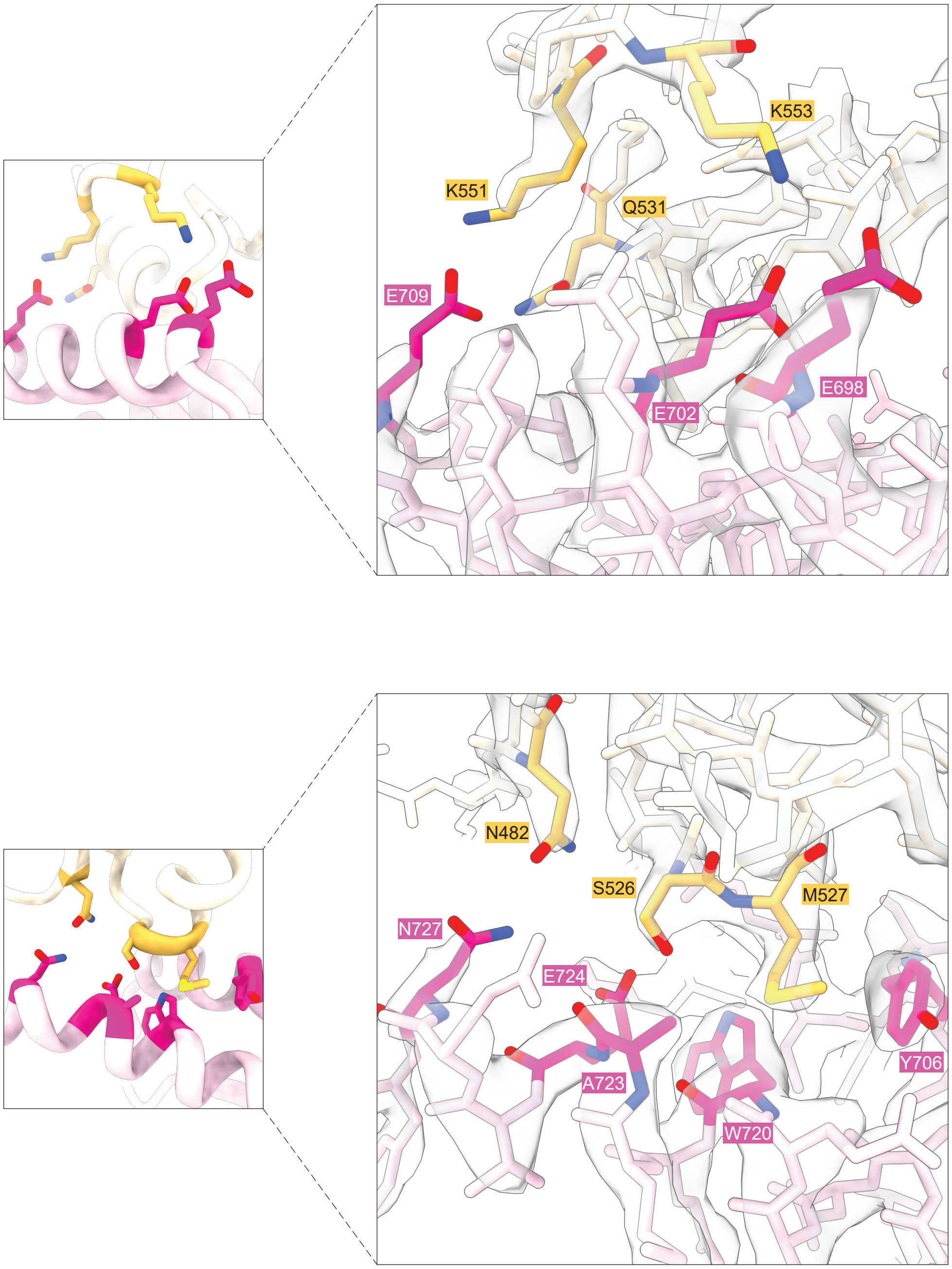

Fig. S16. Csx29-Csx30 interface in the active CASP complex. Interfacing residues, as in Fig. 3A, shown with corresponding EM density.

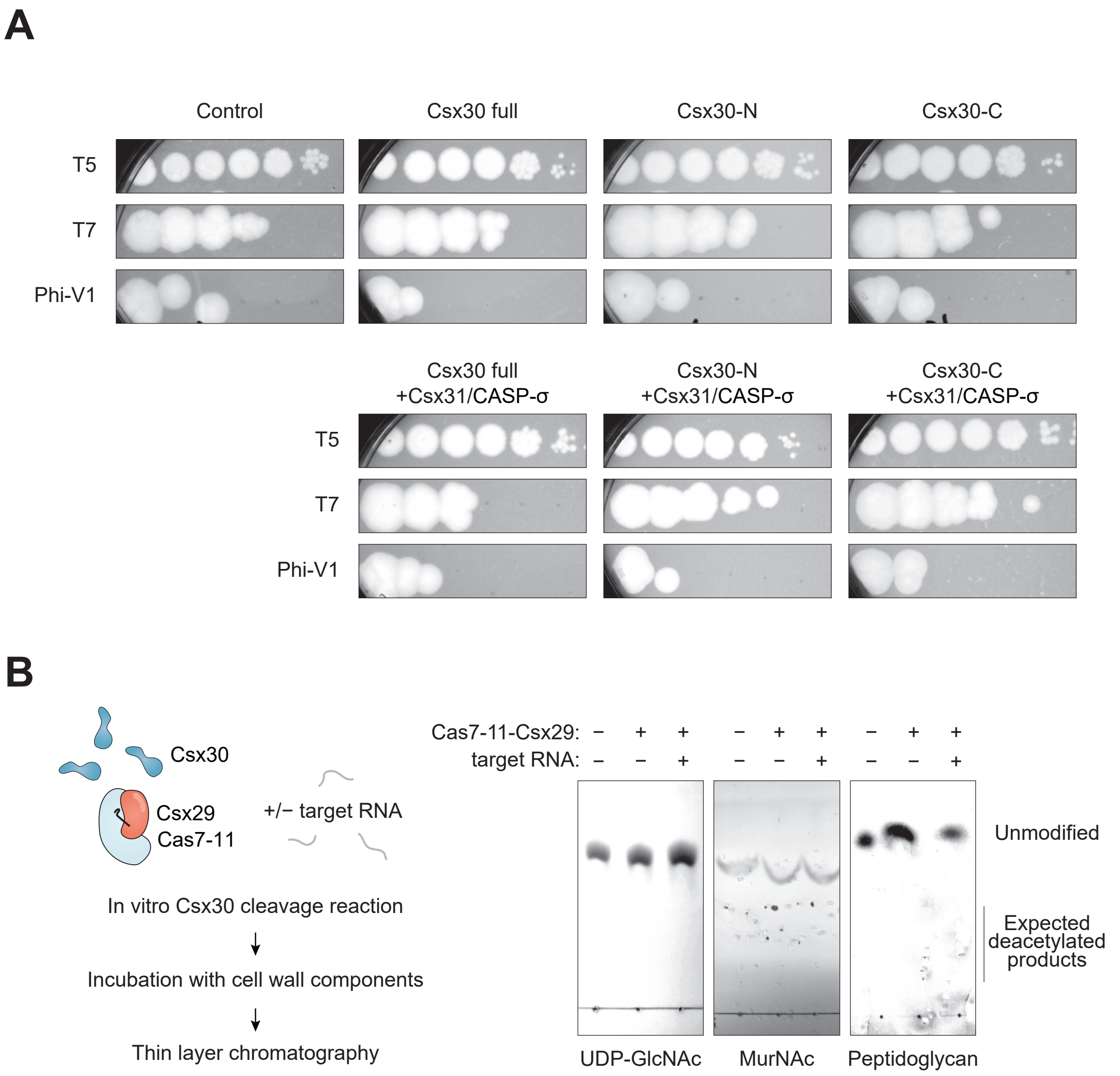

Fig. S17. Investigating potential functions of the cleaved Csx30 fragments. (**A**) Phage plaque assays of *E. coli* expressing full-length Csx30 or processed Csx30 fragments with three lab phage. (**B**) Experimental schematic and thin layer chromatography of cell wall components following in vitro incubation with full-length or cleaved Csx30.

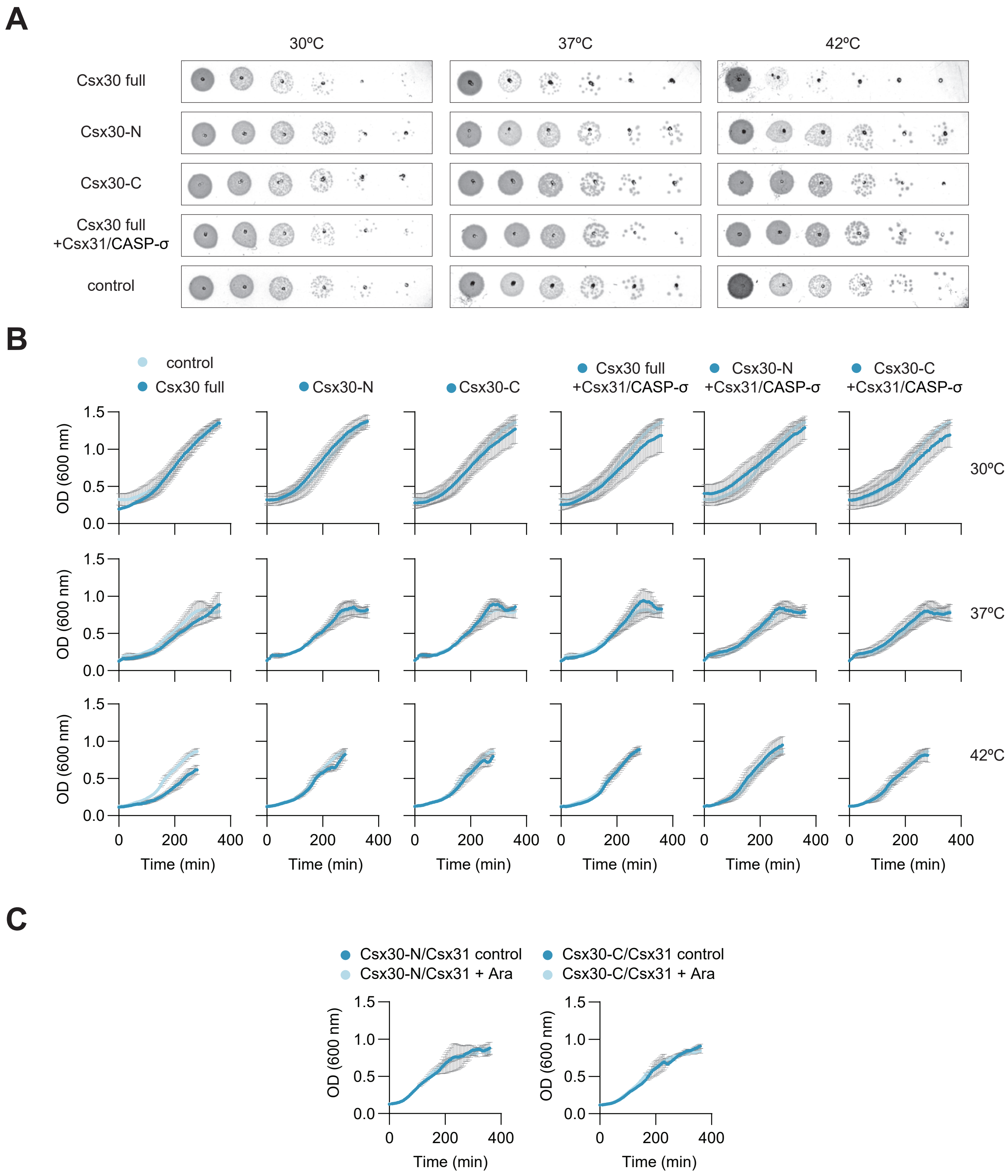

Fig. S18. Effect of Csx30 fragment expression on cell growth. (**A**) Ten-fold dilutions of *E. coli* overexpressing full-length Csx30, Csx30-N, or Csx30-C grown overnight on agar plates at the indicated temperatures. (**B**) Growth curves of *E. coli* cultures overexpressing full-length Csx30, Csx30-N, or Csx30-C at different temperatures. (**C**) Growth curves of *E. coli* cultures overexpressing Csx30-N or Csx30-C in combination with Csx31.

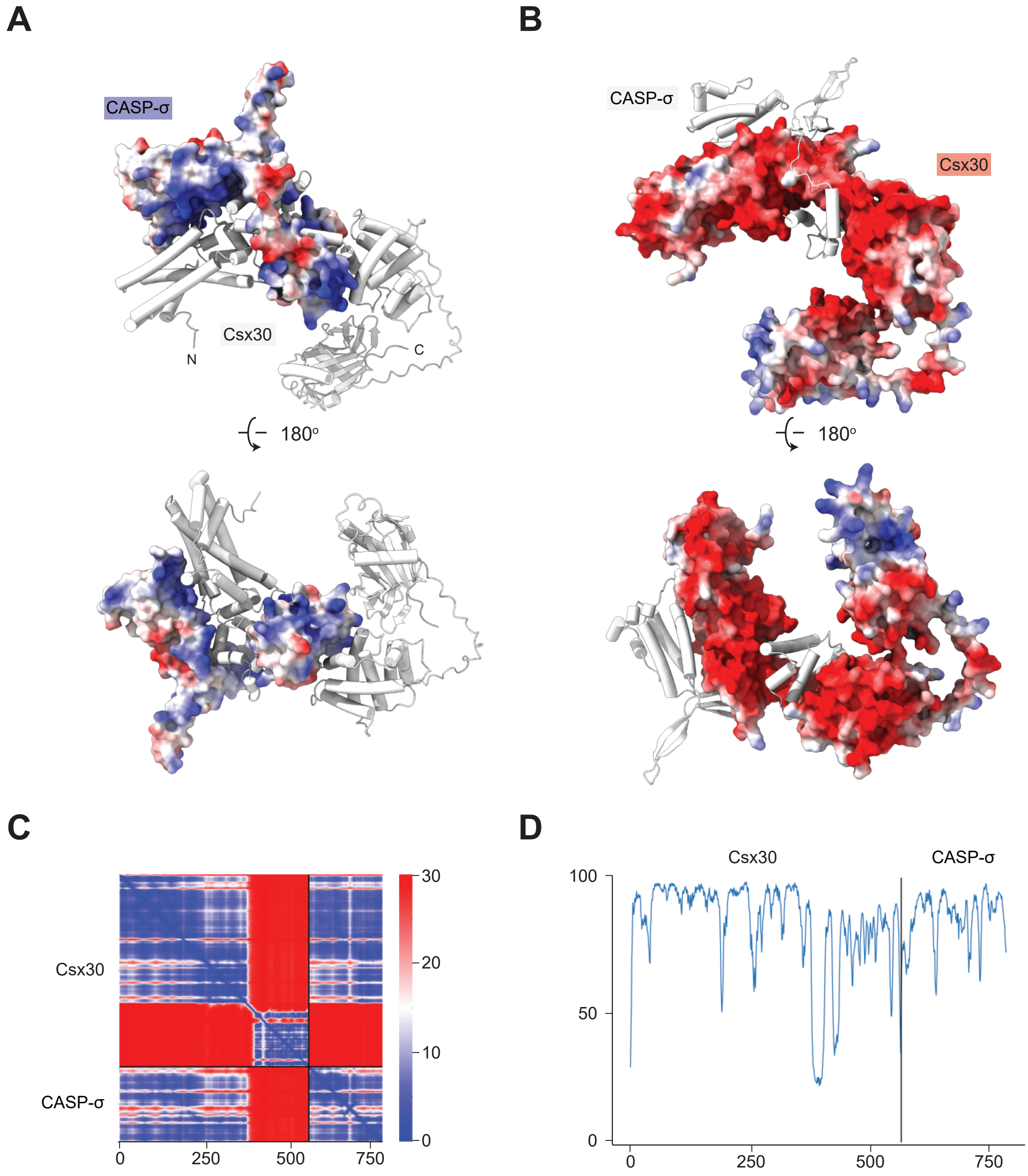

Fig. S19. Computational prediction of a Csx30-CASP-σ complex. (**A**) Coulombic potential of CASP-σ in an AlphaFold2 predicted Csx30-CASP-σ complex. (**B**) Coulombic potential of Csx30 in a AlphaFold2 predicted Csx30-CASP-σ complex. (**C**) Predicted aligned error (PAE) of the predicted Csx30-CASP-σ complex. (**D**) Predicted lDDT-Cα in the predicted Csx30-CASP-σ complex. Charges in panels A and B are shown in a blue (positive) to red (negative) gradient

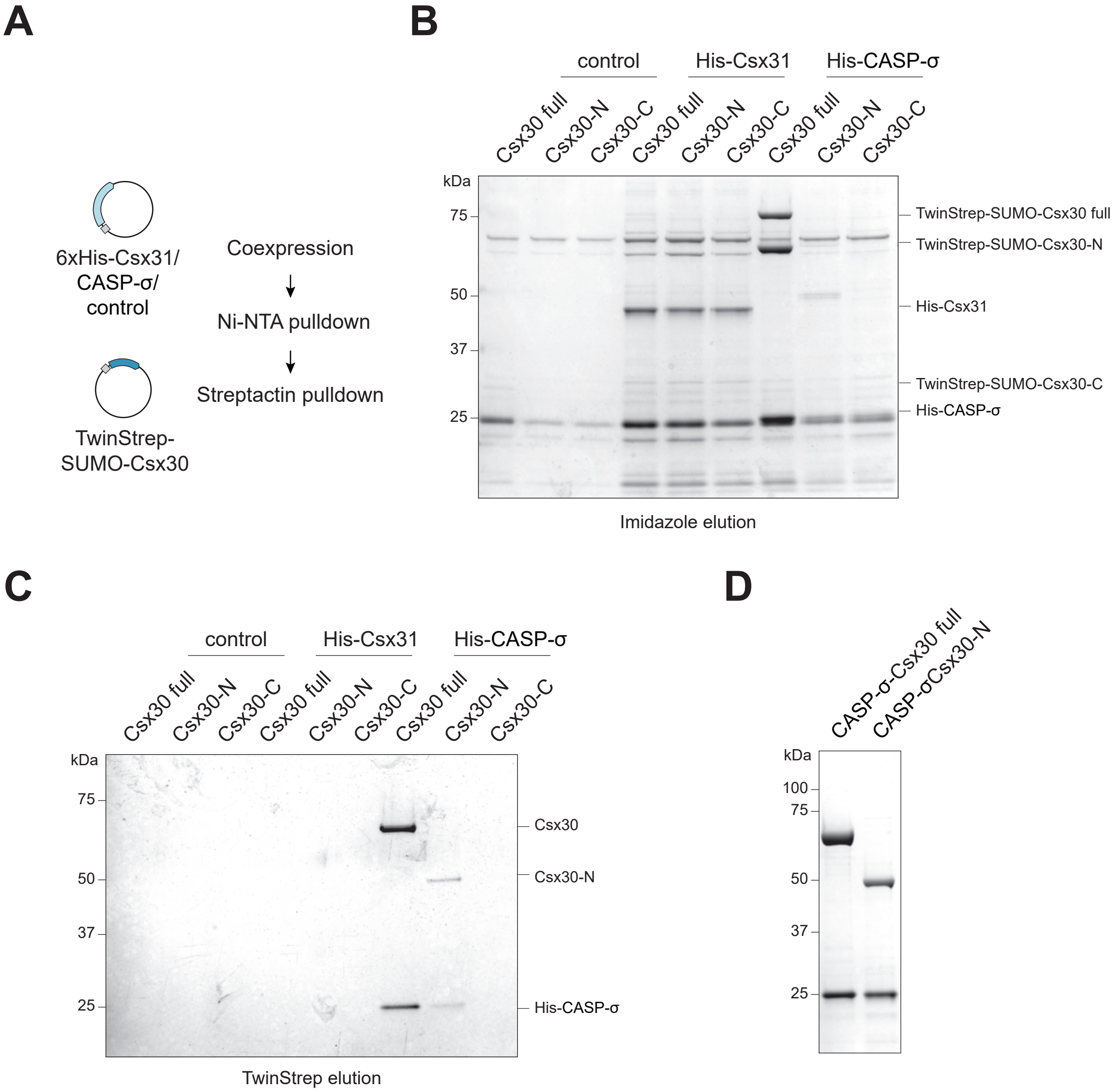

Fig. S20. Physical interaction between Csx30 and CASP-σ. (**A**) Schematic of tandem protein pulldown experiments to identify interactions between Csx30 and Csx31, and Csx30 and CASP-σ. (**B**) Elution from Ni-NTA resin following pulldown of Csx31 and CASP-σ in the presence of full-length Csx30, Csx30-N, or Csx30-C. (**C**) Elution from StrepTactin resin with the SUMO protease Ulp1 yields Csx30-CASP-σ, and a Csx30-N-CASP-σ complex at much lower yield. We did not observe an interaction between Csx30 and Csx31 in similar pulldown experiments. (**D**) Coomassie stained SDS-PAGE of final complexes following protein concentration.

A

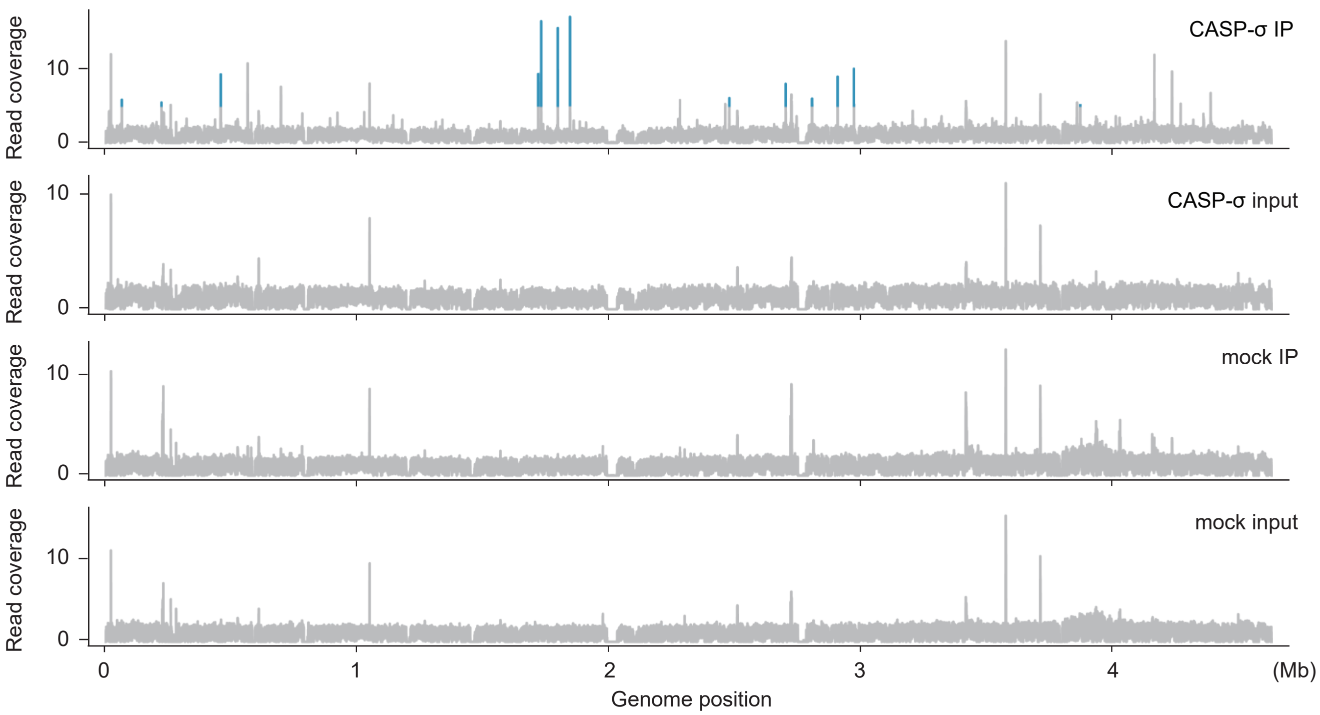

B

Position	Sequence	Fold coverage	Max reads
63,579	GGCACCATTAAGTACGTATGGACCACCAA	5.8	145
221,463	GTTCACAATCGCAGAAACGGTACGATACA	5.4	136
457,787	AGTAACATTCTGCTGATCGGTCCGACCGG	9.2	231
1,721,009	CTTCACAAAGTAGATTCCTTTACGACCGA	9.3	232
1,733,454	AGTAACAATTTGCCGCCAGAAACGACCAC	16.4	410
1,799,888	CGTCACATTCTGACCAAAAAAGCGACCAA	15.5	387
1,848,117	GCTCACAATCCAGACAGTTTCGCGACAAT	17.0	425
2,482,622	ACCCACATTTGACGTGCCGGTACGACAGC	6.0	151
2,707,069	GCTCACATTGACCACGGTAAATCGACGCT	8.0	199
2,812,164	ACCCACATATTGGTGGCTGGAACGACCGC	6.0	149
2,914,012	CGTCACATTCTGTAAGCCATTAAGACGCG	8.9	223
2,978,873	TGCCACATTCACTGTTCAGCACCGATACC	10.0	250
3,880,808	TTTCACAACGAACCCGGATTTTTCACCGC	5.1	127

C

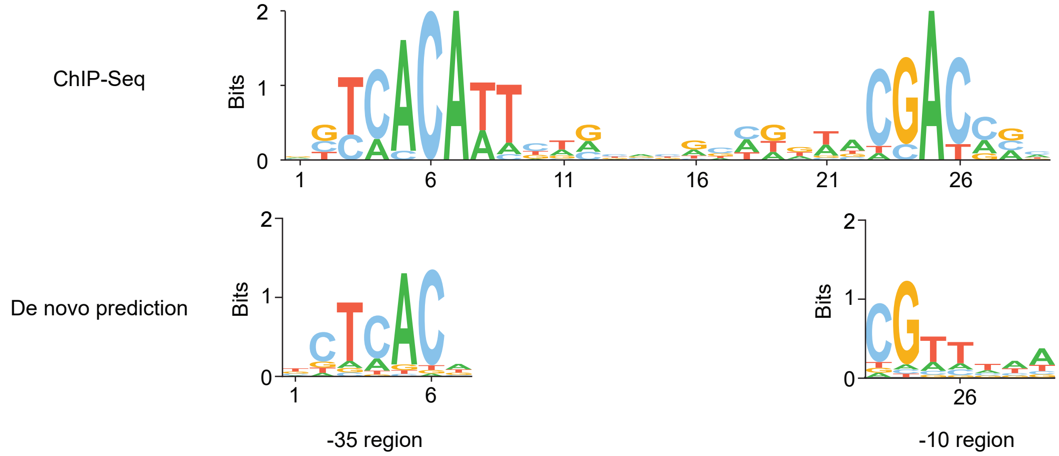

Fig. S21. CASP-σ ChIP-seq analysis in E. coli. (**A**) CASP-σ ChIP-seq reads mapped to the E. coli genome. Significant peaks identified over input and mock IP controls are highlighted in blue. Read coverage was calculated relative to median coverage per sample. (**B**) Alignment of ChIP-seq peaks revealing the presence of a conserved CASP-σ binding motif. (**C**) Comparison of the experimentally determined and computationally predicted CASP-σ binding motif (see methods for details).

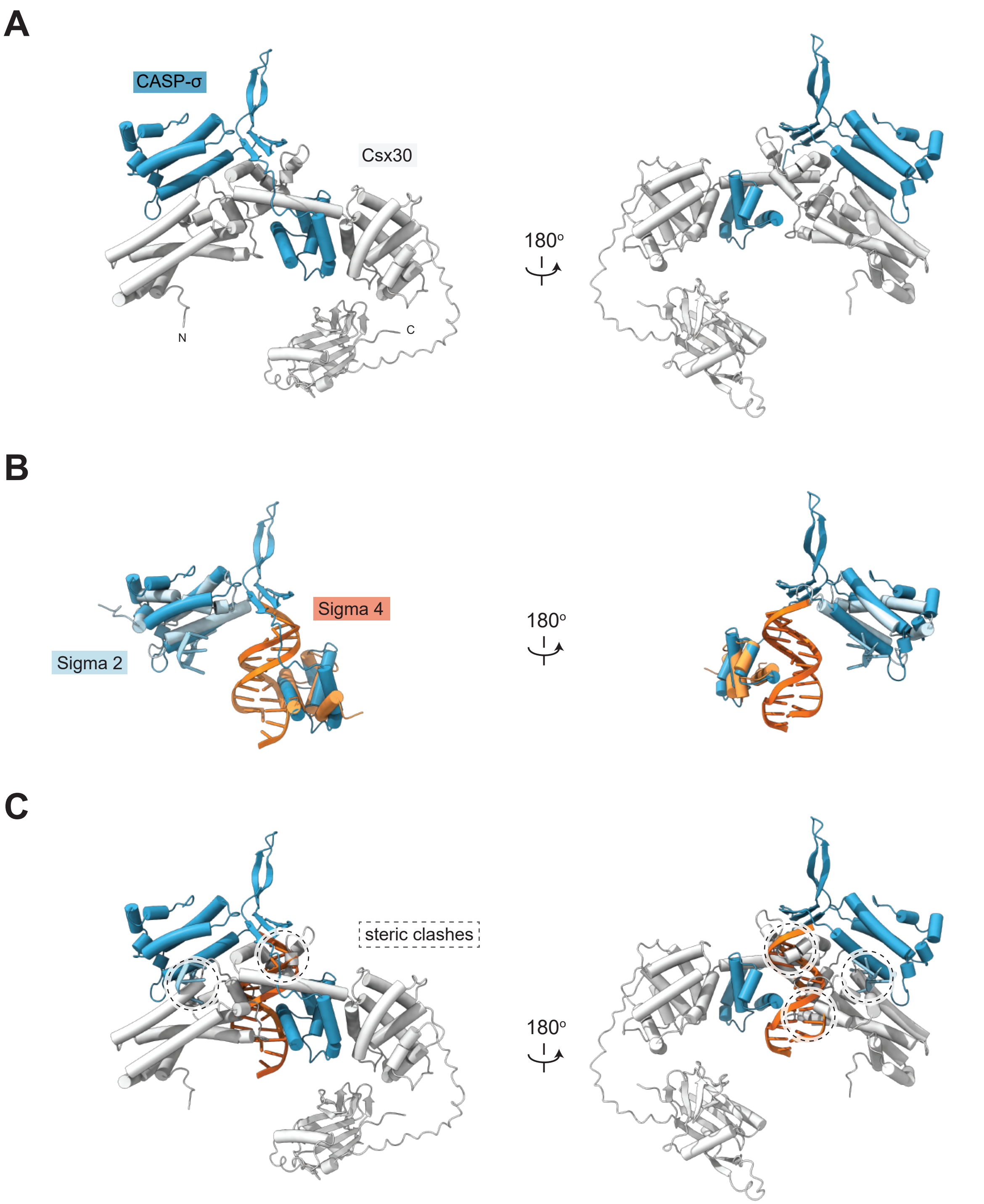

Fig. S22. Computational prediction that the Csx30-CASP-σ interaction blocks CASP-σ DNA binding. (**A**) An AlphaFold2 predicted Csx30-CASP-σ complex. (**B**) Alignment of the predicted CASP-σ structure with experimental structures of the sigma 2 (PDB:5OR5) and sigma 4 domains (PDB:2H27) revealing the position of bound DNA. (**C**) Alignment of the Csx30-CASP-σ complex with modeled sigma-bound DNA highlighting numerous steric clashes.

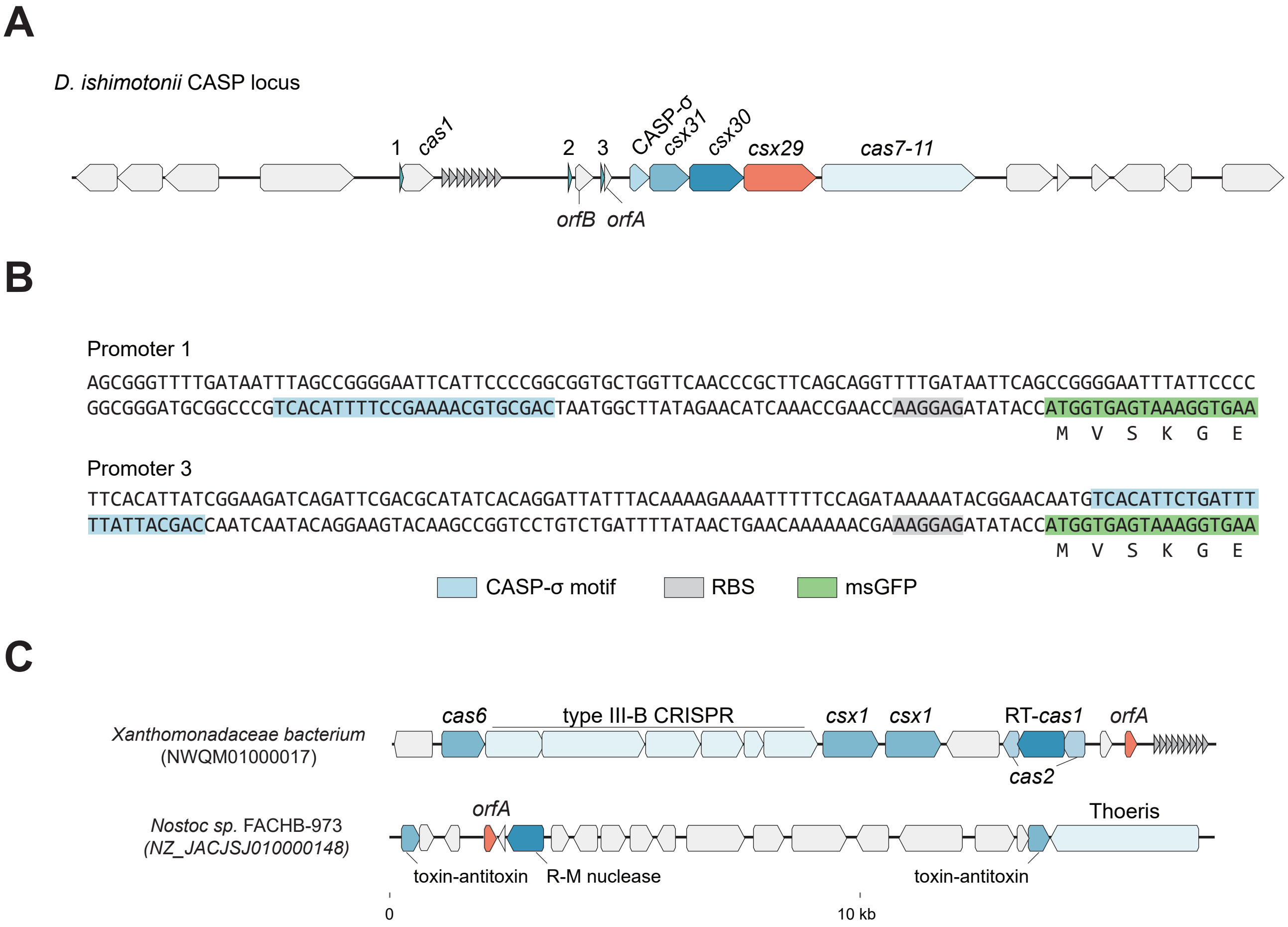

Fig. S23. Predicted transcriptional targets of CASP-σ in *D. ishimotonii*. (**A**) Schematic of the DiCASP locus and three identified CASP-σ motifs. (**B**) Design of transcriptional fluorescent reporters containing CASP-σ motifs. (**C**) Computational identification of *orfA* in a type III-B CRISPR locus and a defense island.

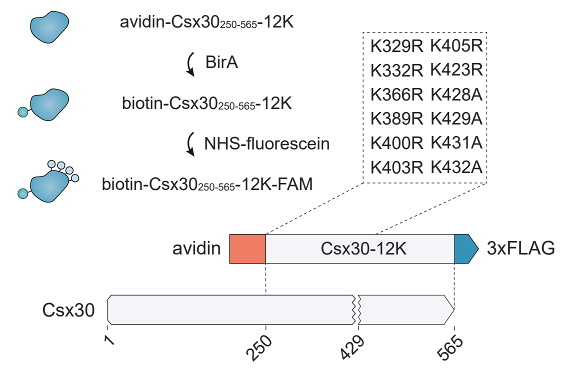

Fig. S24. Schematic of an engineered Csx30 substrate containing residues 250-565 for diagnostic applications and a labeling strategy for generating fluorescent and immobilized Csx30-based substrates. Eight lysine residues in the N-terminal fragment were mutated to arginine to force NHS-FAM labeling of the C-terminal fragment alone. Four lysine residues around the cleavage site were mutated to alanine to prevent NHS-FAM labeling which might block cleavage by Csx29.

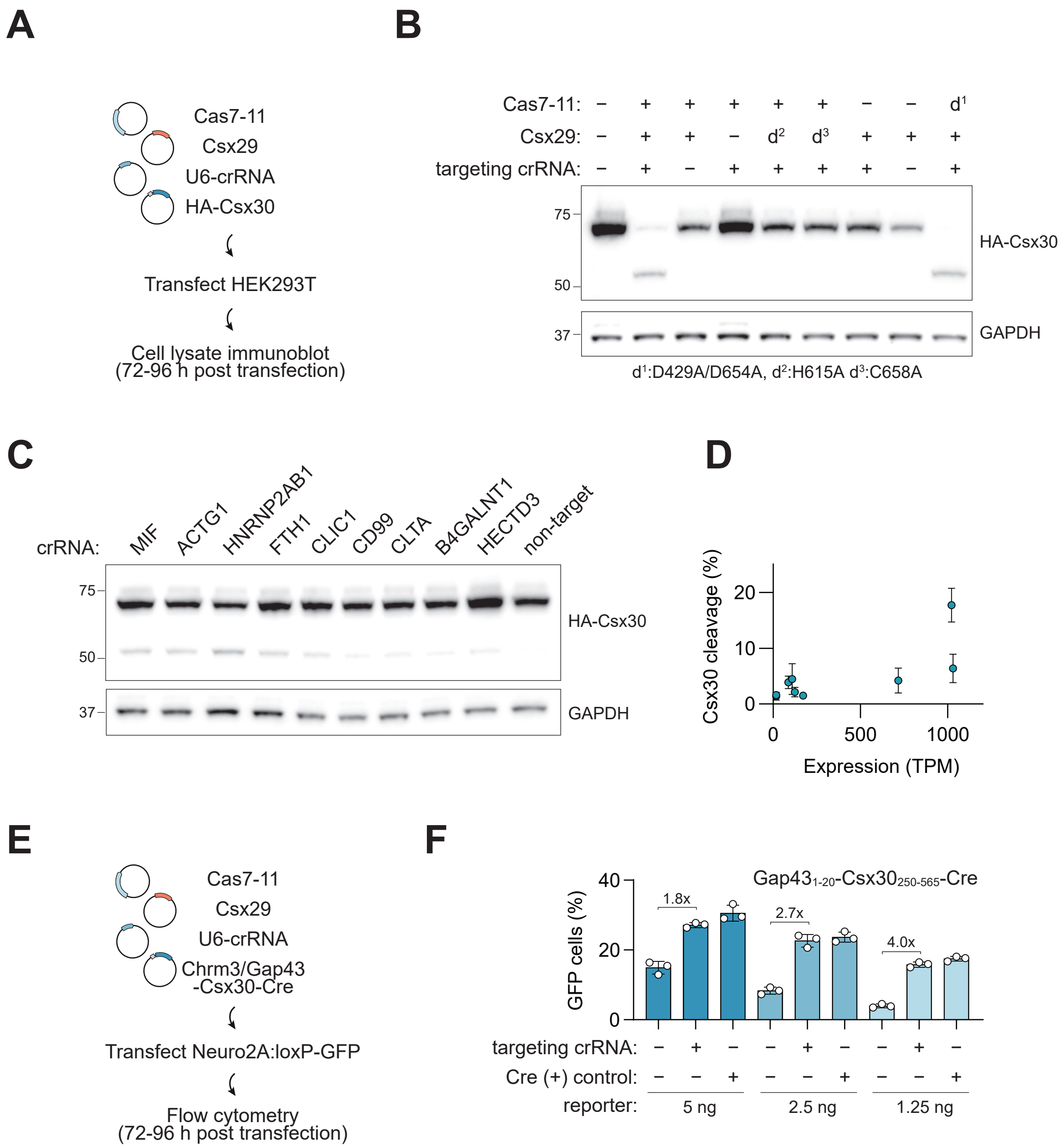

Fig. S25. RNA sensing applications with DiCASP in human cells. (**A**) Schematic of experiments to test Csx30 cleavage in human cells. (**B**) Immunoblot analysis of Csx30 protein cleavage in HEK293T human cells transfected with DiCASP. (**C**) Immunoblot analysis of Csx30 cleavage efficiency using crRNA targeting endogenous RNA transcripts in HEK293T cells. (**D**) Quantification of Csx30 cleavage efficiency versus RNA transcript abundance. RNA expression levels are reported as Transcripts Per Million (TPM). n = 3 replicates, error bars represent standard error of the mean. (**E**) Schematic of experiments to test DiCASP activity and membrane anchored Cre reporteres in mouse Neuro2A cells. (**F**) Flow cytometry of DiCASP activity in Neuro2A:loxP-GFP cells using a growth arrest protein 43 (Gap43) tethered reporter ($Gap43_{1\text{-}20}$-$Csx30_{250\text{-}565}$-Cre). n = 3 replicates, error bars represent standard deviation from the mean. Spacer sequences are listed in table S5.

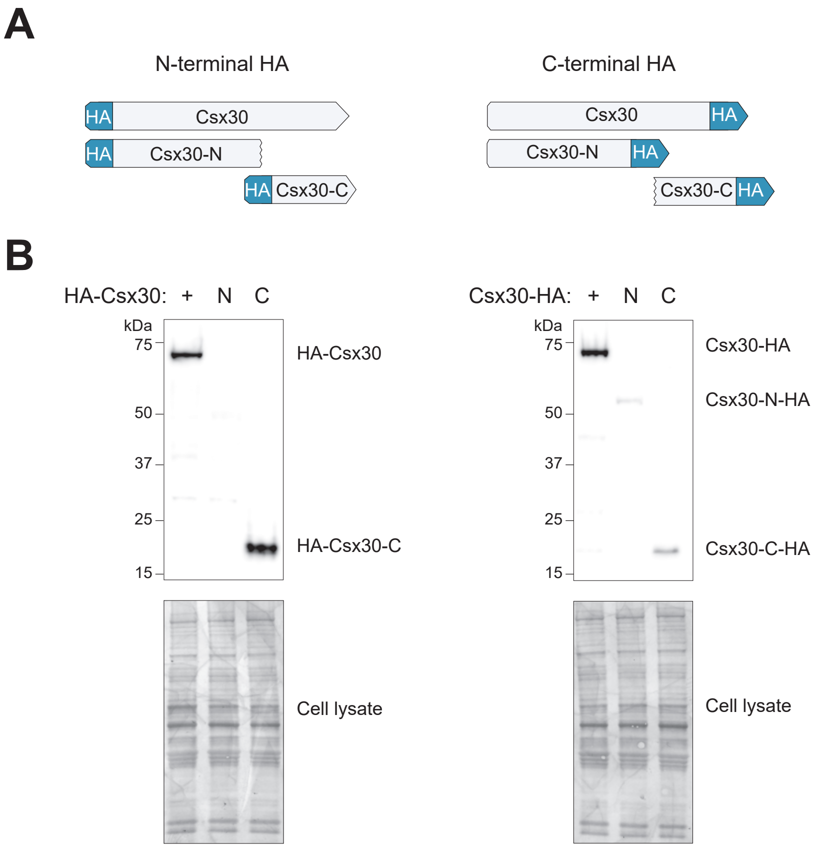

Fig. S26. Expression level of Csx30 fragments in *E. coli*. (**A**) Schematic of N-terminal and C-terminal HA-tagged Csx30 constructs. (**B**) Immunoblot analysis of HA-tagged Csx30 protein levels in *E. coli* and Coomassie stained membranes to show total cell lysate loaded.

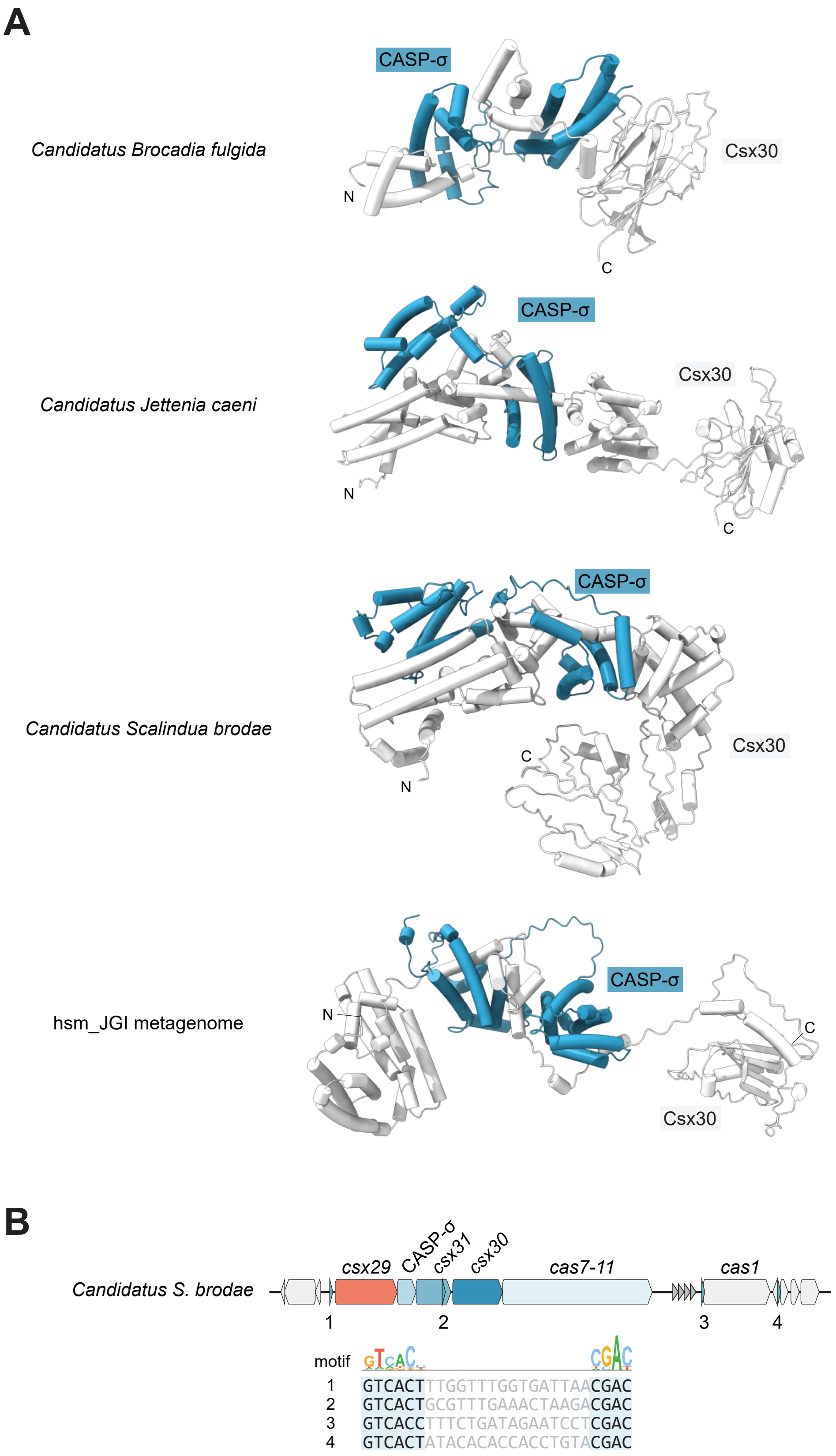

Fig. S27. Predicted CASP-σ inhibition and transcriptional targets in other type III-E CASP systems. (**A**) AlphaFold2 structural predictions of Csx30-CASP-σ binding interactions from additional type III-E CASP loci. (**B**) Predicted binding sites of CASP-σ from *Candidatus Scalindua brodae* using a computationally generated motif.

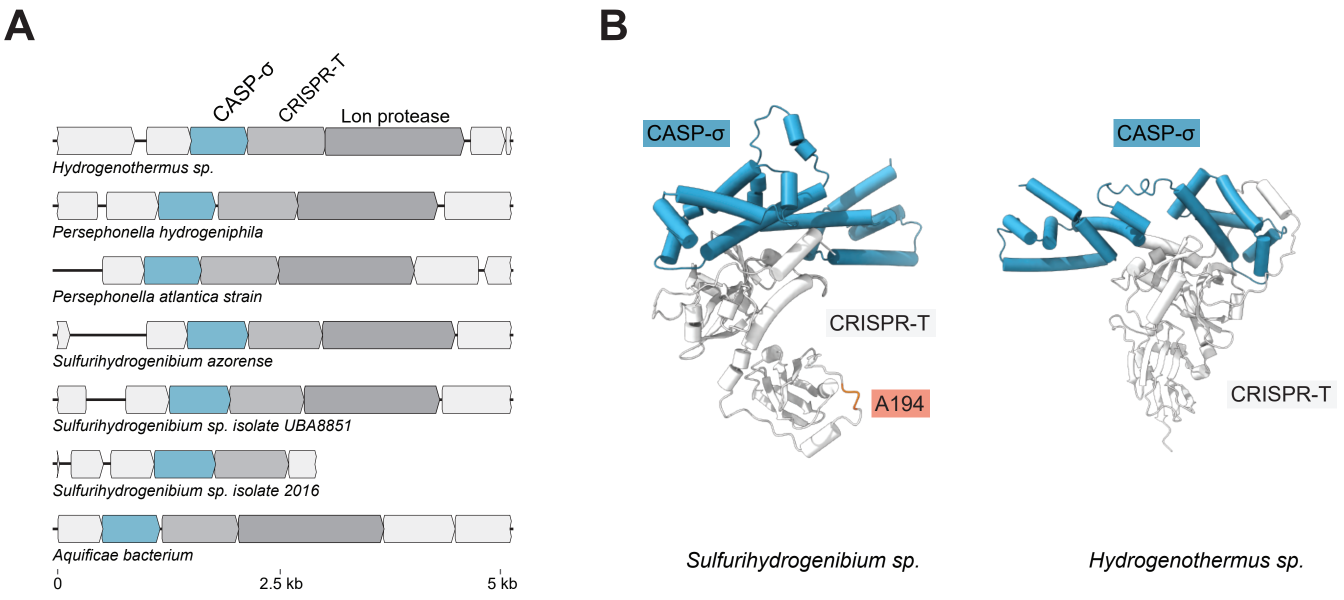

Fig. S28. Predicted sigma factor inhibition in type III CASP Lon systems. (**A**) Schematic of CRISPR-associated Lon protease loci reveals a conserved sigma factor. (**B**) AlphaFold2 structural prediction of a CRISPR-T and sigma factor interaction. The reported cleavage site of CRISPR-T by the Lon protease is highlighted in red (*11*).
